# Intestinal Microbiota Contributes to the Improvement of Alcoholic Hepatitis in Mice Treated With *Schisandra chinensis* Extract

**DOI:** 10.3389/fnut.2022.822429

**Published:** 2022-02-18

**Authors:** Jun-Yan Xiang, Yan-Yu Chi, Jin-Xin Han, Xinyu Shi, Yong Cai, Hongyu Xiang, Qiuhong Xie

**Affiliations:** ^1^Key Laboratory for Molecular Enzymology and Engineering of Ministry of Education, School of Life Sciences, Jilin University, Changchun, China; ^2^School of Life Sciences, Jilin University, Changchun, China; ^3^National Engineering Laboratory for AIDS Vaccine, School of Life Sciences, Jilin University, Changchun, China; ^4^Resources and Applied Microbiology Laboratory, Institute of Changbai Mountain Resource and Health, Jilin University, Fusong, China

**Keywords:** intestinal microbiota, alcoholic hepatitis, *Schisandra chinensis*, short-chain fatty acid, intestinal microbiota transplantation

## Abstract

Alcoholic hepatitis (AH) has a high short-term mortality rate. *Schisandra chinensis* has the potential to ameliorate liver damage and be a source of prebiotics. We aimed to investigate whether *Schisandra chinensis* extract (SCE) can improve AH and the role of the small intestinal and cecal microbiota and their metabolites. UHPLC-QE-MS was used to analyze the chemical components of SCE. The chronic-plus-binge ethanol feeding model was used to induce AH in mice. ^1^H NMR was used to analyze intestinal metabolites. 16S rRNA-based high throughput sequencing was used to evaluate the effects of SCE on intestinal microbiota (IM). Intestinal microbiota transplantation was used to explore the role of IM in SCE treatment of AH. SCE ameliorated AH non-dose-dependently. SCE effectively improved liver inflammation and oxidative/nitrosative stress, strengthened intestinal barrier function, and regulated the composition of IM and the content of short-chain fatty acids (SCFAs) in AH mice. Samples from *in vivo* and *in vitro* SCE-altered IM improved liver status and regulated the IM. The administration of *Lactobacillus plantarum* and *Bifidobacterium breve* ameliorated AH to some extent. The administration of *Enterococcus faecalis* and *Klebsiella oxytoca* had partial beneficial effects on AH. Collectively, IM and metabolites were closely associated with the improvement of SCE on AH. The possible microbe targets were the growth inhibition of *Escherichia-Shigella* and the expansion of SCFA producers, such as *Lactobacillus* and *Bifidobacterium*. *Schisandra chinensis* can be considered as a safe and effective dietary supplement for the prevention and improvement of AH.

## Introduction

Alcoholic hepatitis (AH) is the most common form of alcoholic liver disease (ALD), with a short-term mortality rate of up to 50% (1). Liver steatosis, oxidative stress, cytokine-induced inflammation, destruction of the gut barrier, and alterations in the gut microbiome and metabolome play important roles in the pathogenesis of ALD (2, 3). Short-chain fatty acids (SCFAs), intestinal microbial metabolites that influence intestinal motility and enhance intestinal barrier functions by regulating the function of intestinal epithelial cells, were also reduced (4). These changes lead to increased intestinal permeability and the delivery of bacterial lipopolysaccharide (LPS) into the liver via the portal vein, which activates Kupffer cells to produce inflammatory mediators, such as tumor necrosis factor alpha (TNF-α) and interleukin-1 (IL-1), leading to liver injury (3, 5). There continues to be lack of safe and efficacious drugs for the prevention or treatment of AH (6).

*Schisandra chinensis* is a berry fruit native to Northeast China, Japan, Korea, Manchuria, and the Far East parts of Russia (7). It is often made into a tea or used as a traditional Chinese medicine in Asian countries (8). *Schisandra chinensis* contains a variety of bioactive ingredients with various pharmacological properties (8, 9). Moreover, according to the interpretation of prebiotics by the International Scientific Association for Probiotics and Prebiotics (10), prebiotics are “substrates that are selectively utilized by host microorganisms conferring health benefits.” As *S. chinensis* contains polyphenols and polyunsaturated fatty acids known to exert multiple beneficial health effects, it is likely to be a potential source of prebiotics. *Schisandra chinensis* improved acute alcoholic liver injury by reducing oxidative stress (11), and inhibited inflammatory bowel disease by regulating intestinal microbes and their metabolites (12). However, little is known regarding the relationship between *Schisandra chinensis* extract (SCE)-regulated intestinal microbiota (IM) and AH.

*In vitro* gastrointestinal simulation system can replicate physiological simulation. Usually, the main characteristics of replication are temperature, pH, residence or passing time, and digestive juice composition (13). It is cost effective and free from ethical restrictions (14). It is also used to identify the effects of bacteria and compounds on the gastrointestinal microbiota (15) and evaluate the safety of drug residues (16). Whether the altered IM and its metabolites regulated by drugs in the *in vitro* system can affect the development of the disease remains unclear.

This study investigated the preventive effect of SCE on AH and the role of the IM and its metabolites, SCFAs. To this end, we tested the preventive effect of SCE on AH mice and changes in the IM and SCFAs. SCE was used to alter the IM of mice through the *in vitro* gastrointestinal simulation system and *in vivo* to explore the preventive effects of the altered IM and its metabolites on AH mice. Finally, the possible microbial targets of SCE to prevent AH through IM and the role of SCFA-producing bacteria were explored by the administration of individually cultured bacteria.

## Methods

### *Schisandra chinensis* Extract Preparation

*Schisandra chinensis* (400 g, Liaoning, China) was soaked in 2.5 L of water for 1 h, and boiled for an additional hour, following which the water extracts liquid was separated; these steps were repeated three times. All water extracts were mixed, concentrated to an appropriate volume and lyophilized. The dregs were soaked in 70% ethanol (2.5 L) for 1 h and boiled for an additional hour; the ethanol extract liquid was separated, concentrated to an appropriate volume and lyophilized. Extracts were weighed separately: the water extract was 153.57 g (extraction rate 38.39%), while the ethanol extract was 19.14 g (extraction rate 4.78%).

### Chemical Analyses of *S. chinensis* Extracts

#### Determination of Polysaccharide Content

The total polysaccharide content of both extracts was determined using the phenol-sulfuric acid method. Briefly, sample solution (2, 1, and 0.5 mg/mL; 100 μL) was mixed with phenol solution (5% v/v; 150 μL), following which concentrated sulfuric acid solution (750 μL) was added. The solution was boiled in a water bath for 30 min, and the absorbance was measured at 490 nm. Glucose was used as a reference standard.

#### Determination of Triterpenoid Content

The total triterpenoid content of extracts was determined according to Liu et al. (17) with slight modifications. Briefly, sample solution (1 mg/mL; 75, 150, 200, and 300 μL) was placed in a 70°C water bath until the solvent was completely evaporated and mixed with the vanillin–glacial acetic acid (5% w/v; 40 μL) and 160 μL perchloric acid. The mixture was incubated at 70°C for 20 min, and then 800 μL glacial acetic acid was added and mixed well. The absorbance was measured at 595 nm. Oleanolic acid was used as a reference standard.

#### Determination of Polyphenol Content

The total polyphenol content of extracts was determined according to Adebiyi et al. (18) with slight modifications. Briefly, sample solution (2, 1, 0.5, and 0.25 mg/mL; 10 μL) was mixed with water (590 μL) and 50 μL Folin-Ciocalteu reagent. Na_2_CO_3_ solution (20% w/v; 150 μL) was added and mixed well. Then the mixture was incubated at 40°C in the dark for 30 min, and the absorbance was measured at 750 nm. Gallic acid was used as a reference standard.

#### Determination of Lignins

Lignins were analyzed using High Performance Liquid Chromatography (HPLC). Schisandrol A and schisandrin B (Dalian Meilun Biotechnology Co., Ltd, Dalian, China) were precisely weighed and dissolved in appropriate amount of methanol in volume bottles, respectively, to obtain 1 mg/mL stock solutions of the reference substances. Isovolumetric stock solutions of the reference substances were mixed to obtain 0.5 mg/mL mixed reference solution. *Schisandra chinensis* water extract and ethanol extract were precisely weighed and dissolved in appropriate amount of methanol in volume bottles, respectively, to obtain 10 mg/mL sample solutions. The test solutions were ultrasonic for 20 min and filtered using 0.45 μm film membrane. Chromtographic column: Acchrom XAqua C18 Chromtographic column (4.6 × 250 mm; 5 μm). Chromatographic conditions were based on Xu et al. (19) Flow phase: methanol (A), water (B), gradient elution (0–15 min, 60% A → 75% A; 16–20 min, 75% A; 21–30 min, 75% A → 90% A; 31–40 min, 90%A → 100% A; 41–45 min, 100% A; 46–55 min, 100%A → 60% A); column temperature: 27°C; flow velocity: 0.8 mL/min; detection wavelength: 230 nm.

#### Determination of Organic Acid

Organic acid was analyzed using HPLC. Citric acid (Shanghai Yuanye Bio-Technology Co., Ltd, Shanghai, China) was precisely weighed and dissolved in appropriate amount of water in volume bottles, respectively, to obtain 1 mg/mL stock solution of the reference substance. *Schisandra chinensis* water extract and ethanol extract were precisely weighed and dissolved in appropriate amount of water in volume bottles, respectively, to obtain 10 mg/mL sample solutions. The test solutions were ultrasonic for 20 min and filtered with 0.45 μm film membrane. Chromtographic column: Acchrom XAqua C18 Chromtographic column (4.6 mm × 250 mm; 5 μm). Chromatographic conditions were according to Li et al. (20) Flow phase: acetonitrile and 15 mmol/L KH_2_PO_4_ (1: 99); column temperature: 30°C; flow velocity: 1.0 mL/min; detection wavelength: 210 nm.

#### Determination by UHPLC-QE-MS

UHPLC-QE-MS was performed and analyzed at Biotree Biomedical Technology Co., Ltd, Shanghai, China. First, the samples were extracted: 100 mg of sample was added to 1,000 μL extracted solution (methanol:water = 4:1 and ultrapure water, both containing 1 μg/mL of internal standard), then the mixture was homogenized at 45 Hz for 4 min and followed using ultrasonic for 5 min on an ice-water bath. After placing 1 h in −20°C, the samples were centrifuged at 12,000 rpm for 15 min at 4°C. Finally, the supernatant was carefully filtered through a 0.22 microporous membrane and placed in a fresh 2 mL tube for LC-MS/MS analysis. The quality control sample was prepared by mixing an equal aliquot of the supernatants from all the samples. LC-MS/MS analysis was performed on a thermo scientific ultra-high performance liquid chromatography vanquish system with a Waters UPLC BEH C18 column (2.1 × 100 mm, 1.7 μm). The flow rate was set at 0.4 mL/min and the sample injection volume was set at 5 μL. The mobile phase consisted of 0.1% formic acid in water (A) and 0.1% formic acid in acetonitrile (B). The multi-step linear elution gradient program was as follows: 0–3.5 min, 95–85% A; 3.5–6 min, 85–70% A; 6–6.5, 70–70% A; 6.5–12 min, 70–30% A; 12–12.5 min, 30–30% A; 12.5–18 min, 30–0% A; 18–25 min, 0–0% A; 25–26 min, 0–95% A; 26–30 min, 95–95% A.

An Q Exactive Focus mass spectrometer coupled with an Xcalibur software was employed to obtain the MS and MS/MS data based on the IDA acquisition mode. During each acquisition cycle, the mass range was from 100 to 1,500, and the top ten of every cycle were screened and the corresponding MS/MS data were further acquired. Sheath gas flow rate: 45 Arb, Aux gas flow rate: 15 Arb, Capillary temperature: 400°C, Full ms resolution: 70000, MS/MS resolution: 17500, Collision energy: 15/30/45 in NCE mode, Spray Voltage: 4.0 kV (positive) or −3.8 kV (negative).

### Network Pharmacology Analysis

Target prediction of bioactive components of *S. chinensis* was performed at https://www.tcmsp-e.com/ and http://www.uniprot.org/. Screening related genes of alcoholic liver diseases was performed at https://www.genecards.org/, https://go.drugbank.com/ and https://www.disgenet.org/. Venn diagram was drawn at http://www.bioinformatics.com.cn/. KEGG enrichment analysis and visualization were performed at https://metascape.org/.

### *In vitro* Gastrointestinal Simulation System

*In vitro* methods (PBET: physiologically based extraction test, SHIME^®^: simulator of human intestinal microbial ecosystem) were used according to the method of Wang et al. (21) with slight modifications. Briefly, there were 5 phases from the beginning to the end: stomach, temporary platform, small intestine, cecum, and drain. The inoculum was added, respectively, to the small intestinal and cecal samples of mice at a ratio of sample weight (g): inoculum (mL) = 1:5, and were separately inoculated into the small intestine phase and the cecum phase. The mixtures were incubated in a water bath at 37°C for 24 h and stirred at a low speed (80 rpm). No other manipulations were made during the first 24 h. Afterwards, 20 g feed mixed with 200 mL gastric juice (pH 2.0), was added to the stomach phase and stirred at 37°C for 1 h. Subsequently, the chyme was transferred to the temporary platform phase where the pH was adjusted to 7.0 using saturated NaHCO_3_, and bile salts and pancreatin were added. Then, the mixture was transferred to the small intestine phase, stirred and kept at 37°C for 4 h, and the pH was kept at 7.0 ± 0.2. After that, part of the solution was transferred to the cecum phase, stirred and kept at 37°C, and the pH was kept at 5.7 ± 0.2. Later, the time and speed of transfer were set to allow part of the solution in the cecum phase to drain automatically. The experiment lasted for 32 days. During the initial 24 h, only microbial samples were inoculated. Feed was given twice a day for 15 days, and then SCE was given (only once a day) on the basis of feed for 15 days. On the last day, the solutions in the small intestinal phase and cecal phase were centrifuged at 10,000 rpm for 10 min individually, and the precipitates were added with a protective agent (skim milk powder, trehalose, and VC-sodium) at the ratio of 1: 0.6, quickly and fully mixed, and then lyophilized for storage, and tagged. The experiment was carried out in two rounds. In the first round, 99.9999% N_2_ was used to maintain an anaerobic environment, while in the second round, 10% CO_2_ and 90% N_2_ were used, since it has been reported that CO_2_ can be fixed to form acetate or propionate (22), and can regulate pH and facilitate the growth of some anaerobic bacteria (23). The system was fully flushed with gas before inoculation and after feeding. Samples in the first round were tagged as PS1S and PS1C, those in the second round were tagged as PS2S and PS2C. The last letter “S” represented the small intestinal phase and “C” represented the cecal phase.

### Animal Study

Five different animal studies were conducted. Eight-week-old C57BL/6J male mice purchased from Liaoning Changsheng Biotechnology Co., Ltd. (Benxi, China) were housed (four mice per cage) in a temperature- and humidity-controlled facility (individual ventilated cages) with free access to water and standard diet. Following adaptation to the environment for 2 days, the mice were randomly divided into groups with 12 mice in each group and then adapted to the Lieber DeCarli control liquid diet for 7 days. All mice were gavaged for 20 days (vehicle/supplement), and on day 11 modeling [chronic-plus-binge ethanol feeding model (24)] was initiated. The Lieber DeCarli control liquid diet was given the same amount (isocaloric ration) as Lieber DeCarli alcoholic liquid diet (5% v/v ethanol). In the morning of the 11th day of modeling, mice fasted overnight were gavaged with a 5-g/kg dose of ethanol or isocaloric maltose. After 9 h, the mice were anesthetized with ether and sacrificed for specimens. Liquid feed was purchased from Trophic Animal Feed High-tech Co. Ltd (Nantong, China). Lieber DeCarli control liquid diet: 35% fat, 18% protein, 47% carbohydrate. Lieber DeCarli ethanol liquid diet: 35% fat, 18% protein, 19% carbohydrate, 28% ethanol. Mice in groups beginning with “C” were fed with Lieber DeCarli control liquid diet, and mice in groups beginning with “AH” were fed with Lieber DeCarli ethanol liquid diet.

#### Pre-experiment

Five groups of mice were included in the pre-experiment (8 mice/group): (1) the CON group (health control); (2) the AH group (disease control); (3–5) the AH-SCL, AH-SCM, AH-SCH groups, mice were received low, medium, and high doses of SCE, respectively. Pharmacopeia of the people's republic of China (2015) records that the daily dose of humans for *S. chinensis* (raw medicine) is 2–6 g. According to the description of the conversion method of the drug dose between humans and animals in “Medical Laboratory Animal Science,” the dose of human was converted into the dose of mice: 6 g raw medicine per day for a 60 kg person is equivalent to 0.9 g/kg raw *S. chinensis* per day for mice, which is a medium dose. The doses of water or ethanol extracts were calculated by multiplying the raw *S. chinensis* dose by the extraction rate, which were 0.3455 and 0.0430 g/kg, respectively. Half of the medium dose was considered as a low dose, and twice the medium dose was considered as a high dose. The water and the ethanol extracts were dissolved/suspended together in distilled water for administration.

#### Round One

Four groups of mice were included in this round (12 mice/group): (1–2) the CON and AH group; (3–4) the C-SC and AH-SC groups, mice were administered with SCE. Medium dose administration was used in this round.

#### Round Two

A total of 168 mice were included in this round (12 mice/group, and 60 donor mice): (1–2) the CON and AH group; (3) the AH-P group, mice were received the protective agent; (4–7) the AH-PS1S, AH-PS1C, AH-PS2S, and AH-PS2C groups, mice were administered with *in vitro* samples, respectively; (8–9) the AH-SI and AH-Cc group, mice were received SCE-altered small intestinal and cecal content of healthy mice, respectively. The mice used to provide intestinal samples *in vivo* were given SCE every day, the dose of which was the same as Round-One animal experiment. Ten days after administration, three mice were sacrificed per day for collecting contents in the small intestine and the cecum. Equal weight of the two contents was suspended, respectively, in saline and given intragastrically to mice in AH-SI and AH-Cc groups.

#### Round Three

Seven groups of mice were included in this round (12 mice/group): (1–2) the CON and AH group; (3–6) the AH-KO, AH-EF, AH-LP, and AH-BB groups, mice were received *Klebsiella oxytoca* CGMCC1.3718, *Enterococcus faecalis* CICC20427, *Lactobacillus plantarum* RAM0303, and *Bifidobacterium breve* CICC6182, respectively; (7) the AH-LB groups, mice were received *L. plantarum* and *B. breve*. 5 × 10^8^ CFU strains were given to the mouse per day.

#### Additional Experiment

Five groups of mice were included in the additional experiment (12 mice/group): (1–2) the CON and AH group; (3) the AH-CMC group, mice were received 0.5% carboxymethyl cellulose sodium (CMC); (4) the AH-SolA group, mice were administered with schisandrol A and 0.5% CMC; (5) the AH-SchB group, mice were administered with schisandrin B and 0.5% CMC. Based on the percentage of peak areas of schisandrol A and schisandrin B in the fingerprint of ethanol extract and their doses given to mice by gavage in published articles, 15 mg/kg schisandrol A or 7 mg/kg schisandrin B with 0.5% CMC were given to mice.

All animal experiments were approved by the Experimental Animal Welfare and Ethics Committee of School of Life Sciences, Jilin University (permit NO. 2018SY0312, 2018SY0506, 2018SY1117, 2019SY0425, and 2019SY0529) and were in full compliance with all relevant animal welfare guidelines and legislation.

### Histological Analysis

Part of the liver, ileum, and colon samples were fixed in 4% formalin and embedded in paraffin, then processed for staining with hematoxylin-eosin and performed the immunohistochemical test (occludin, ileum only). Samples of 3–4 mice in each group were randomly selected for histological analysis. Anti-occludin antibody (Cat NO., 66378; Lot, 10019234) was purchased from Proteintech (Chicago, USA).

### Assay Kits

Plasma concentration of LPS was determined using a Mouse Lipopolysaccharides ELISA Kit (CUSABIO Biotech Co., Ltd., China). Alanine aminotransferase assay kit, Aspartate aminotransferase assay kit, Superoxide Dismutase (SOD) assay kit (WST-1 method), Nitric Oxide Synthase (NOS) typed assay kit (Colormetric method) and Triglyceride assay kit were from Nanjing Jiancheng Bioengineering Institute, China. Samples of 8–12 mice in each group were randomly selected for the assays.

### Quantitative Real-Time Polymerase Chain Reaction (qRT-PCR)

RNA extraction and reverse transcription were performed on samples of liver and small intestine, followed by qRT-PCR. Experimental methods and conditions were based on previously published protocols (25). Primer sequences used in this study are listed in Supplementary Table 6. The 2^−ΔΔCt^ method was applied to calculate the fold change of relative gene expression. Samples of 6–8 mice in each group were randomly selected for qRT-PCR.

### Western Blotting Analysis

Experimental methods were based on previously published protocols (25). For the extracted ileal protein, 30 μg was applied to detect the amount of β-actin protein, and 90 μg was applied to detect the amount of occludin protein. Samples of 6–8 mice in each group were randomly selected for western blotting analysis. Anti-occludin antibody (Cat NO., AB167161; Lot, GR119583-32) was purchased from Abcam (Cambridge, UK).

### Intestinal Microbiota Analysis

Samples (small intestinal contents or cecal contents) of mice in the same cage were mixed as one sample for testing (i.e., *n* = 3). Total bacterial DNA was extracted from samples using the Power Soil DNA Isolation Kit (MO BIO Laboratories) according to the manufacturer's protocol. DNA quality and quantity were assessed by the ratios of 260/280 nm and 260/230 nm. Then DNA was stored at −80°C until further processing. The V3-V4 region of the bacterial 16S rRNA gene was amplified with the common primer pair (Forward primer, 5'-ACTCCTACGGGAGGCAGCA-3'; reverse primer, 5'-GGACTACHVGGGTWTCTAAT-3') combined with adapter sequences and barcode sequences. PCR amplification was performed in a total volume of 50 μL, which contained 10 μL Buffer, 0.2 μL Q5 High-Fidelity DNA Polymerase, 10 μL High GC Enhancer, 1 μL dNTP, 10 μM of each primer and 60 ng genome DNA. Thermal cycling conditions were as follows: an initial denaturation at 95°C for 5 min, followed by 15 cycles at 95°C for 1 min, 50°C for 1 min and 72°C for 1 min, with a final extension at 72°C for 7 min. The PCR products from the first step PCR were purified through VAHTSTM DNA Clean Beads. A second round PCR was then performed in a 40 μL reaction which contained 20 μL 2 × Phusion HF MM, 8 μL ddH_2_O, 10 μM of each primer, and 10 μL PCR products from the first step. Thermal cycling conditions were as follows: an initial denaturation at 98°C for 30 s, followed by 10 cycles at 98°C for 10 s, 65°C for 30 s, and 72°C for 30 s, with a final extension at 72°C for 5 min. Finally, all PCR products were quantified using Quant-iT™ dsDNA HS Reagent and pooled together. High-throughput sequencing analysis of bacterial rRNA genes was performed on the purified, pooled sample using the Illumina Hiseq 2500 platform (2 × 250 paired ends) at Biomarker Technologies Corporation, Beijing, China.

### Short-Chain Fatty Acids Analysis

The concentration of lactic acid and SCFAs in a mixed sample of the small intestine contents or a single sample of the cecal contents was analyzed using high performance liquid chromatography (HPLC). Experimental methods and HPLC conditions were based on previously published protocols (26). Samples of at least 3 mice in each group were randomly selected for SCFA analysis.

### ^1^H Nuclear Magnetic Resonance (NMR) Metabolomics Analysis

For small intestinal or cecal contents, the sample was mixed in the same way as in the intestinal microbiological analysis (i.e., *n* = 3). ^1^H NMR spectra of samples were collected at 298 K on an AVANCE III 600 MHz spectrometer. The NMR spectrum was recorded using the water-presaturated standard one-dimensional NOESY pulse sequence. The free induction decays were collected with 128 transient into 32 k data points using a spectral width of 10 kHz with a relaxation delay of 3 s, and relaxation time (2 nτ) of 100 ms.

### Antimicrobial Assay and Experiments on Bacterial Growth

We conducted bacteriostatic and growth experiments on *Salmonella enterica subsp. Enterica serovar Typhimurium* CICC10420, *Enterobacter cloacae* CICC21539, *Vibrio cholerae* CICC23795, *Staphylococcus aureus* RAM0410, *Pseudomonas aeruginosa* (P7) RAM0501, *Pseudomonas aeruginosa* (P11) RAM0502, *Pseudomonas aeruginosa* (P16) RAM0503, *Vibrio parahaemolyticus* CICC23924, *Shigella flexneri* RAM0510, *Klebsiella oxytoca* CGMCC1.3718, *Lactobacillus rhamnosus* RAM0305, *Lactobacillus salivarius* RAM0306, *Lactobacillus plantarum* RAM0303, *Lactobacillus casei* RAM0601, *Lactobacillus paracasei* RAM0602, *Lactobacillus bulgaricus* RAM0102, *Lactobacillus reuteri* RAM0101, *Streptococcus thermophiles* RAM0403, *Enterococcus faecium* CICC20536, *Enterococcus faecalis* CICC20427, *Bifidobacterium adolescentis* CICC6178, *Bifidobacterium bifidum* CICC6169, *Bifidobacterium longum* CICC6197, and *Bifidobacterium breve* CICC6182.

#### Antibacterial Activity Test

SCE was tested using disc diffusion test. A suspension (100 μL) containing 1.0 × 10^6^ CFU/mL of bacteria was spread on a nutrient agar medium. Sterilized disc papers (diameter 6 mm) were impregnated with 30 μL of SCE (the amount on each disc was equivalent to the daily dose of a 25 g mouse) and placed onto the medium. Control was prepared using sterilized water instead of essential oil. The inoculated plates were incubated at 37°C for 24 h. The diameter of the clear zone around the disc was measured and expressed in millimeters as its antibacterial activity. Three disks per plate were used and each test was run in triplicate.

#### Bacterial Growth Promotion Test

Inoculated the bacteria to be tested into a 5 mL tube supplemented with 3 mL liquid culture medium for cultivation. Cells were passaged at 24 h intervals and strains grown to log phase at the fourth passage were used as back-up cultures. Three milliliters of liquid medium and low, medium, and high doses of the three extracts (ethanol extract, water extract, and mixed extract) and 30 μL back-up cultures were added to 5 mL tubes and incubated anaerobically at 37°C. Colony-Forming Units (CFU) was measured at 2-h intervals for 0–14 h after inoculation and growth curves were prepared.

### Statistical Analysis

Data were represented as the mean ± SD. One-way analysis of variance with LSD *post-hoc* test was used for multiple comparisons; *p* < 0.05 was considered statistically significant. GraphPad Prism 8 was used for graphing, and SPSS 26.0.0.2 was used for statistical analysis. The heatmap and network of Spearman correlation were drawn using R language and Cytoscape V.3.9.0. Image Studio 4.0 and Quantity One 4.6.2 were used for western bolt analysis. Image Pro Plus 6.0 was used for immunohistochemical analysis and length measurement. Histogram of species distribution was drawn on www.biocloud.net. Adobe Illustrator 24.0.3 was used for final figure editing.

## Results

### Main Constituents of SCE

The main constituents of SCE include phenols, flavonoids, terpenoids, lignans, phenylpropanoids, amino acid derivatives, alkaloids, carbohydrates and derivatives, and organic acids and derivatives (Supplementary Figures 1, 2, Supplementary Table 1, Supplementary Material 1). Some of the main components of SCE and their biological activities are shown in Supplementary Material 2. Flavonoids have been widely investigated for their anti-cancer, antioxidant, and anti-inflammatory activities. The main phenylpropanoids and lignans in SCE have been studied to demonstrate their hepatoprotective effects. In addition, amino acids, vitamins, succinic acid, and other substances with intestinal benefits were also detected in SCE. From the main components we detected, those with an oral bioavailability >20% and drug-like properties >0.18 were screened and processed by network pharmacology analysis (Supplementary Figure 3). There are 138 potential gene targets of *S. chinensis* for the treatment of ALD, and the pathways involved PI3K-Akt signaling pathway, TNF signaling pathway, non-alcoholic fatty liver disease, and nuclear factor kappa-B (NF-κB) signaling pathway.

### Effects of SCE on Alcohol-Induced Liver Injury, Lipid Accumulation, Inflammation, Alcohol Metabolism, and Intestinal Functions

Since the two extracts possess similar chemical compositions, we mixed the two extracts for administration. In animal pre-experiment, we investigated the effects of different doses of SCE in AH mice. ALT and AST levels in the serum, TNOS and iNOS activities and inflammatory cytokine expression in the liver were determined, and it was found that the improvement of AH by SCE was not dose-dependent and the best effect was obtained at a medium dose (Supplementary Figure 4). Therefore, a medium dose was used for the following study.

**Figure 1:**
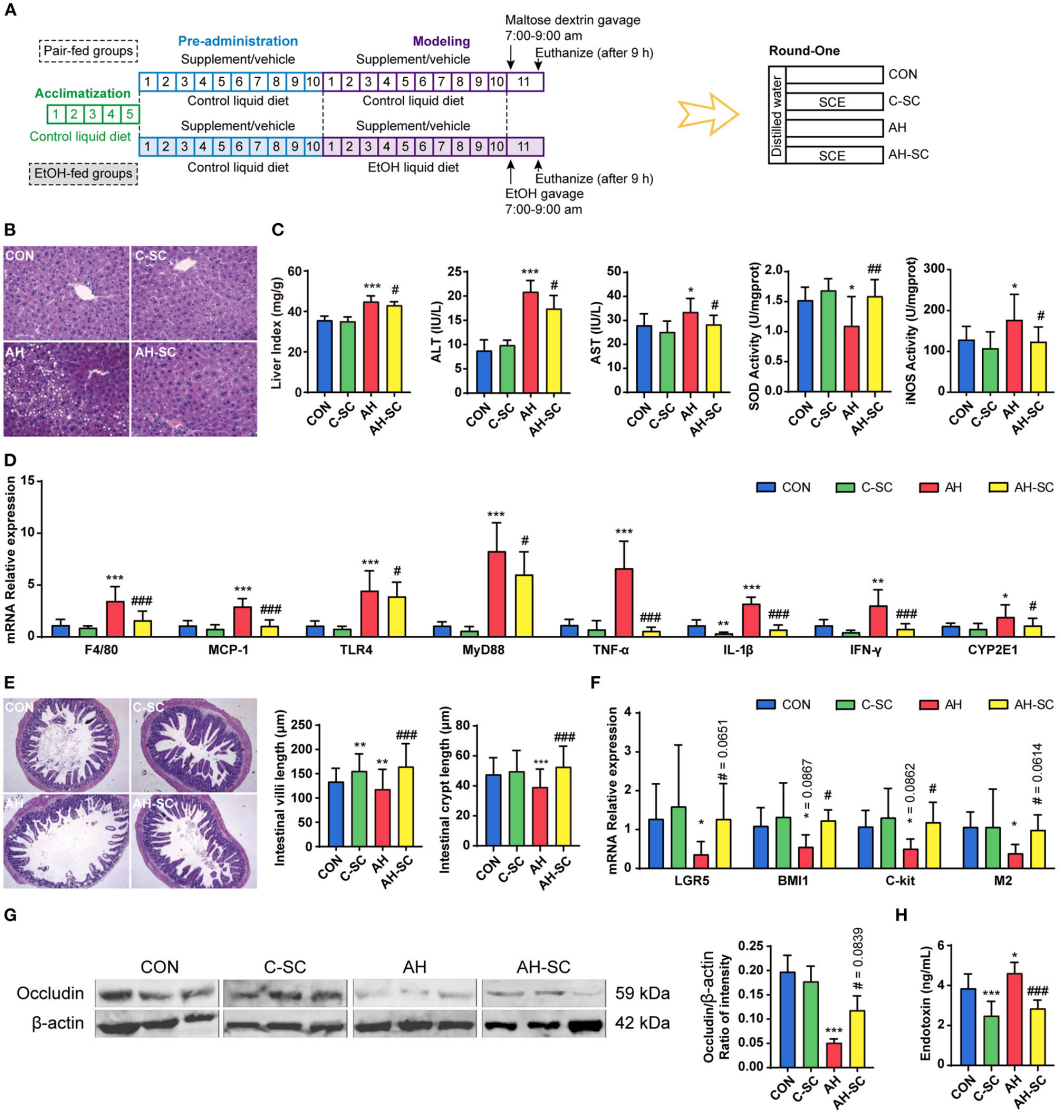
Effects of SCE on alcohol-induced liver injury, lipid accumulation, inflammation, alcohol metabolism, and intestinal functions. **(A)** Process of animal study and administration details in each group (*n* = 12 mice/group). Mice in groups beginning with “C” were fed with Lieber DeCarli control liquid diet, and mice in groups beginning with “AH” were fed with Lieber DeCarli ethanol liquid diet. **(B)** Hepatic H&E staining (magnification: 400 ×). **(C)** Liver index, ALT and AST in serum, SOD, and iNOS activities in the liver. **(D)** Hepatic mRNA expression of *F4/80, Mcp-1, Tlr4, Myd88, Tnf-*α, *Il-1*β, *Ifn-*γ, and *Cyp2e1*. **(E)** Small intestinal H&E staining (magnification: 100 ×) and the length of villi and crypts in the small intestine. **(F)** Small intestinal mRNA expression of *Lgr5, Bmi1, C-kit*, and *M2*. **(G)** Western blot for occludin in the ileum. **(H)** Serum LPS level. Results were shown as the mean ± SD. *p < 0.05, **p < 0.01, ***p < 0.001 compared with CON group, and #*p* < 0.05, ##*p* < 0.01, ###*p* < 0.001 compared with AH group by ANOVA one-way statistical analysis.

Ethanol-induced liver injury was characterized by serum alanine aminotransferase (ALT) release, inflammatory infiltration, and hepatocyte lipid accumulation (27). The process of supplement and modeling, and grouping information is shown in Figure 1A. We observed that SCE administration significantly prevented abnormal accumulation of lipids in the liver, inhibited the increase in liver index, serum ALT, and aspartate aminotransferase (AST), resisted the decrease of superoxide dismutase (SOD) activity and the increase of inducible nitric oxide synthase (iNOS) activity, and suppressed the high expression of liver inflammatory cytokines compared with the AH group (Figures 1B–D). Additionally, alcohol consumption significantly enhanced the expression of enzymes related to alcohol metabolism, and SCE significantly inhibited the increase in *Cyp2e1* expression (Figure 1D, Supplementary Figure 5). CYP2E1 is not only an enzyme related to alcohol metabolism, it also plays a role in a key step in the production of large amounts of reactive oxygen species (2). Although SCE had a limited effect on alcohol metabolism, the results demonstrated that SCE administration inhibited oxidative stress. SCE well-improved the liver status of AH, and regulated liver oxidative stress by regulating the activity of related enzymes and inhibiting relevant gene expression. SCE had little effect on the liver status of the healthy mice.

Alcohol can destroy intestinal epithelial integrity, thereby increasing intestinal permeability, which releases bacterial-derived endotoxin into the circulation (28). Intestinal stem cells and tight junction proteins are essential components of intestinal barrier, which is also influenced by intestinal motility (29). Hematoxylin and eosin (H&E) staining of the small intestine showed that alcohol intake severely damaged the villi structure of the small intestine, which was largely prevented by SCE administration (Figure 1E). The alcohol-related reduction in the expression of *Lgr5* and *Bmi1*, which are important markers of intestinal stem cells (30), was inhibited by SCE (Figure 1F). SCE administration prevented the alcohol-induced significant decrease in the expression of *C-kit* and *M2*, which regulate intestinal motility (Figure 1F) (31, 32). SCE administration substantially inhibited the significant reduction of small intestinal occludin (western blot) caused by alcohol intake (Figure 1G). Additionally, LPS in serum was measured to evaluate the release of endotoxin into the circulation, and it was observed to decrease after SCE administration (Figure 1H). SCE improved intestinal barrier function by acting on intestinal stem cells, the small intestine structure, tight junction proteins, and intestinal motility. The results of serum LPS level also reflected the enhancement of the intestinal barrier caused by the drug. SCE had little effect on the intestinal status of the healthy mice.

### Effects of SCE on IM, Metabolites, and SCFAs and Their Correlation With Host Parameters, as Well as on Bacterial Growth *in vitro*

**Figure 2:**
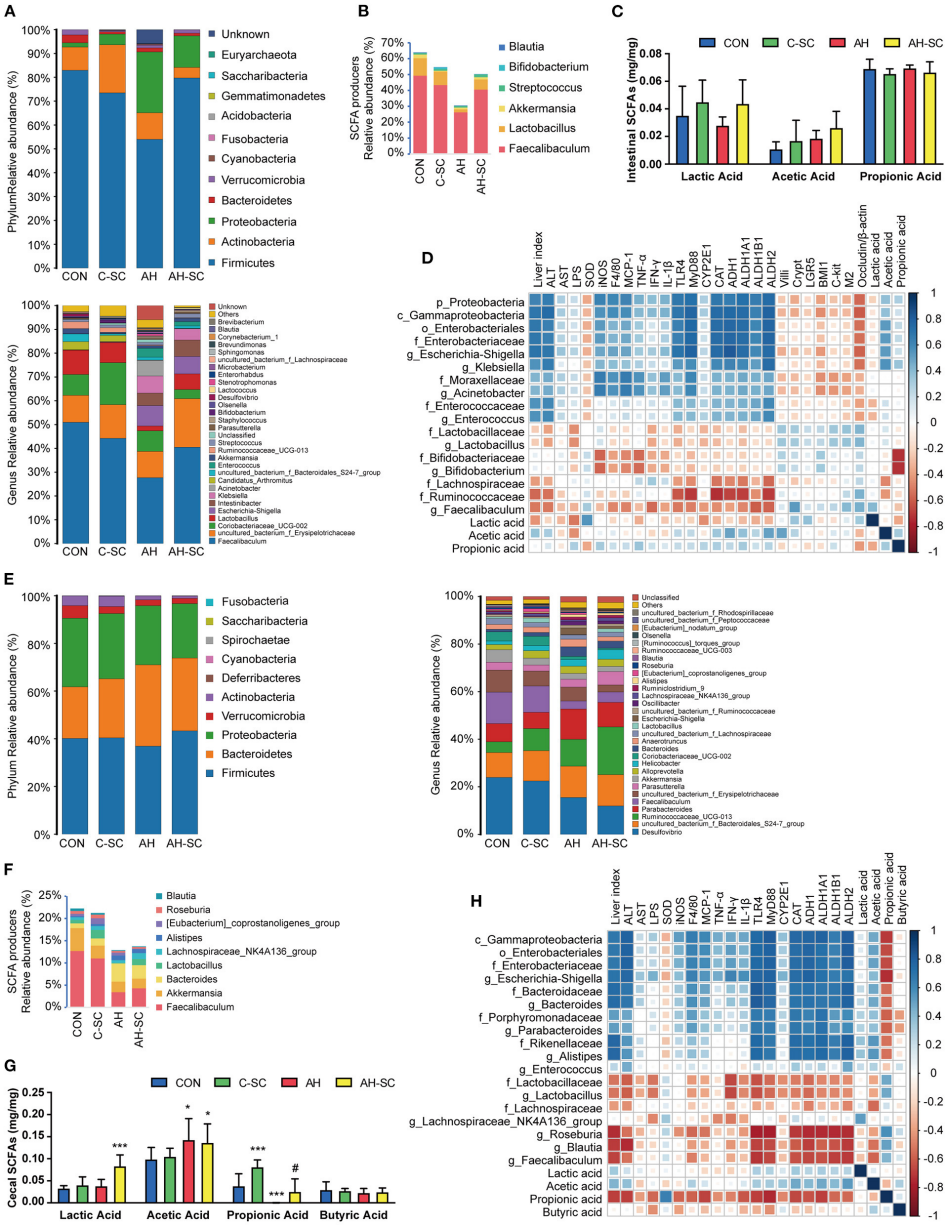
Effects of SCE on IM and SCFAs and their correlation with host parameters. **(A)** Relative abundance of microbiota at phylum and genus levels, **(B)** relative abundance of SCFA producers, **(C)** SCFA content in the small intestine. **(D)** Spearman correlation of small intestinal microbiota and host parameters. **(E)** Relative abundance of microbiota at phylum and genus levels, **(F)** relative abundance of SCFA producers, **(G)** SCFA content in the cecum. **(H)** Spearman correlation of cecal microbiota and host parameters. Results **(C,G)** were shown as the mean ± SD. *p < 0.05, ***p < 0.001 compared with CON group, and #*p* < 0.05 compared with AH group by ANOVA one-way statistical analysis.

Gut microbes can affect intestinal barrier function (27). The overall effects of SCE administration on microbiota in the small intestine and cecum were evaluated, and the contents of SCFAs were determined (Figure 2, Supplementary Figure 6). In the small intestine (Figure 2A), alcohol intake resulted in changes in microbial composition, with a decrease in Firmicutes and an increase in Proteobacteria. SCE precluded this trend. The main changes in Firmicutes involved *Faecalibaculum* and *Lactobacillus*. The main changes in Proteobacteria involved *Escherichia-Shigella, Klebsiella*, and *Acinetobacter*, which are all members of Gammaproteobacteria. The total proportion of SCFA-producing bacteria was reduced in the AH group and rebounded in the AH-SC group (Figure 2B). Lactic acid levels were decreased by alcohol intake but increased with SCE. Acetic acid levels increased with alcohol intake (Figure 2C). In the cecum (Figure 2E), alcohol intake and SCE supplementation did not cause significant changes in microbial composition. SCE inhibited the abnormal growth of Enterococcaceae, *Escherichia-Shigella, Parabacteroides, Bacteroides*, and *Alistipes* and the lessened abundance of *Faecalibaculum* and *Lactobacillus* caused by alcohol intake. SCE had no obvious effect on the total proportion of SCFA producers (Figure 2F). The lactic acid level was the highest in the AH-SC group. Alcohol intake significantly increased the acetic acid content. Propionic acid was significantly decreased after alcohol intake, while SCE significantly increased it (Figure 2G). Additionally, the metabolic changes of the small intestine and cecum (^1^H NMR) in the model used in this study were mainly in amino acids, but were not obvious overall (Supplementary Tables 2, 3, see details in “Supplementary Material_main.pdf”).

The potential correlation between changes in microbiota, metabolites in different intestinal segments, and liver injury, intestinal barrier functions in the host was assessed using Spearman correlation analysis. In the small intestine (Figure 2D), Proteobacteria was correlated with aggravating AH, among which *Escherichia-Shigella, Klebsiella*, and *Acinetobacter* were prominent. *Enterococcus* was also correlated with AH exacerbations. *Lactobacillus, Bifidobacterium, Faecalibaculum*, Lachnospiraceae, Ruminococcaceae, and lactic acid were associated with ameliorating AH. Acetic acid was positively correlated with the dominant bacteria in Proteobacteria. In the cecum (Figure 2H), a large part of the changes in bacterial abundance was associated with alcohol intake and significantly associated with acetic acid content. Gammaproteobacteria (mainly *Escherichia-Shigella*), *Bacteroides, Parabacteroides*, and *Alistipes* were associated with deteriorating AH and negatively correlated with propionic acid. *Lactobacillus, Faecalibaculum*, and Lachnospiraceae (*Roseburia* and *Blautia*) were associated with alleviating AH and positively correlated with propionic acid. Propionic acid was associated with improving AH.

Additionally, we demonstrated in *in vitro* experiments that SCE selectively inhibited bacterial growth (Supplementary Tables 4, 5, see details in “Supplementary Material_main.pdf”).

### Effects of SCE on Intestinal Microbiota Composition and SCFAs in an *in vitro* Gastrointestinal Simulation System

**Figure 3:**
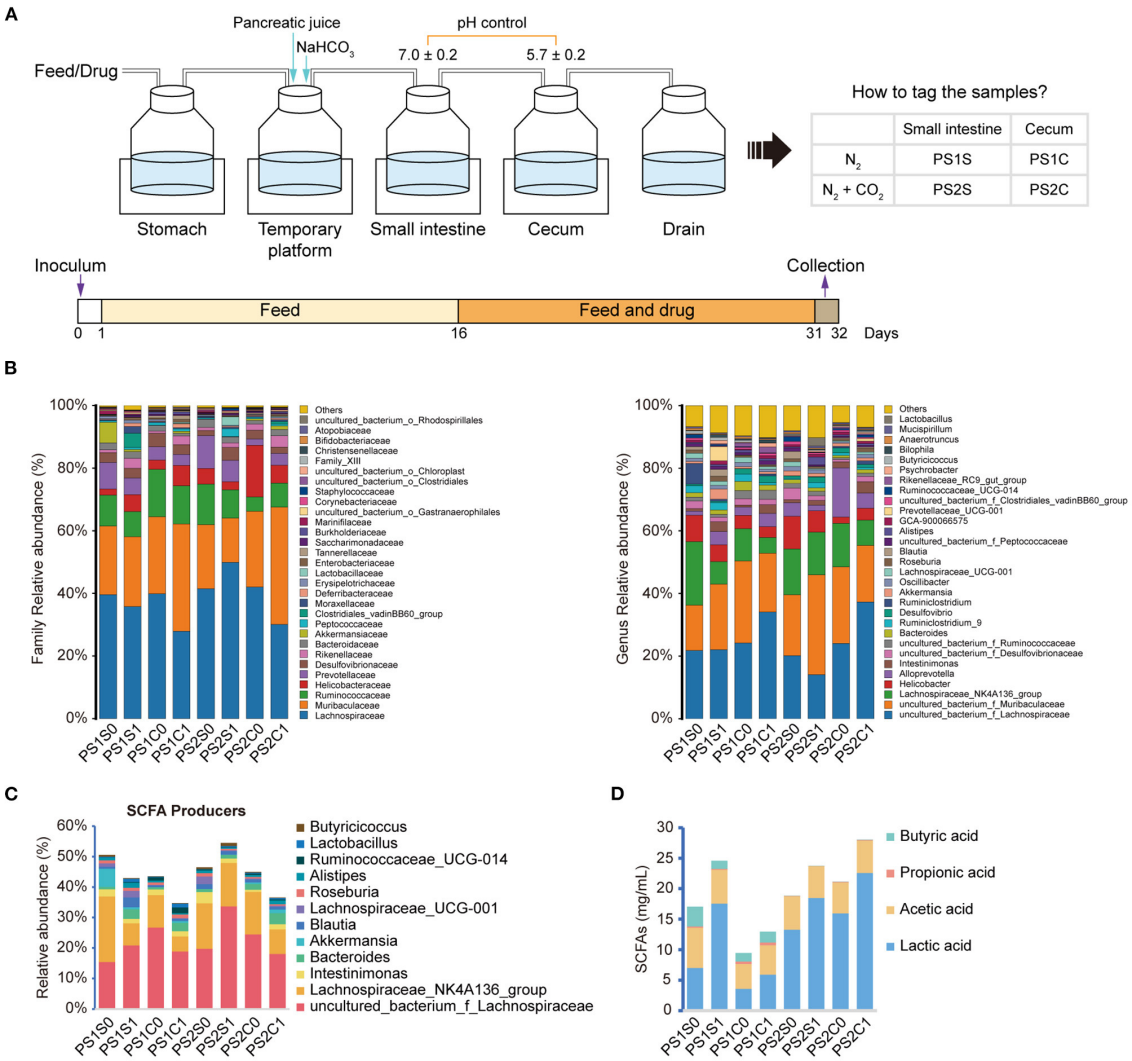
Effects of SCE on intestinal microbe composition and SCFAs in an *in vitro* gastrointestinal simulation system. **(A)** Process of *in vitro* gastrointestinal simulation system and group information. **(B)** Relative abundance of microbiota at family and genus levels. **(C)** Relative abundance of SCFA producers. **(D)** SCFA content. **(B–D)** Groups with “0” at the end represents samples before administration with SCE, groups with “1” at the end represents samples after administration.

In round-one animal experiment, we observed that SCE was effective in improving AH, and it regulated the IM and SCFAs in mice and selectively inhibited bacterial growth *in vitro*. To determine the role of IM and its metabolites in improving AH, and whether they were altered by drug stimulation and body influence, or assist in modulating disease states, we first observed the effects of SCE on the IM and SCFAs *in vitro* via samples of small intestinal and cecal content collected from healthy mice (Figure 3, Supplementary Figure 7). Group information was shown in Figure 3A. At the phylum level, the proportion of Firmicutes and Bacteroides changed in each group (Supplementary Figure 7). At the genus level, the main change was that each group had a new component ratio of SCFA producers (Figures 3B,C). While measuring the SCFA content (Figure 3D), we observed that the lactic acid content increased markedly in all groups, and the propionic acid content increased only in PS1C. In general, the effects of SCE on the IM and SCFAs in the *in vitro* system were some extent different from those *in vivo*. The changes in proportions in the anaerobic environment maintained by N_2_, rather than CO_2_ and N_2_, were more similar to the changes observed *in vivo*.

### Effects of SCE-Altered *in vivo*/*vitro* IM and Its Metabolites on Alcohol-Induced Liver Injury, Lipid Accumulation, Inflammation, and Intestinal Functions

**Figure 4:**
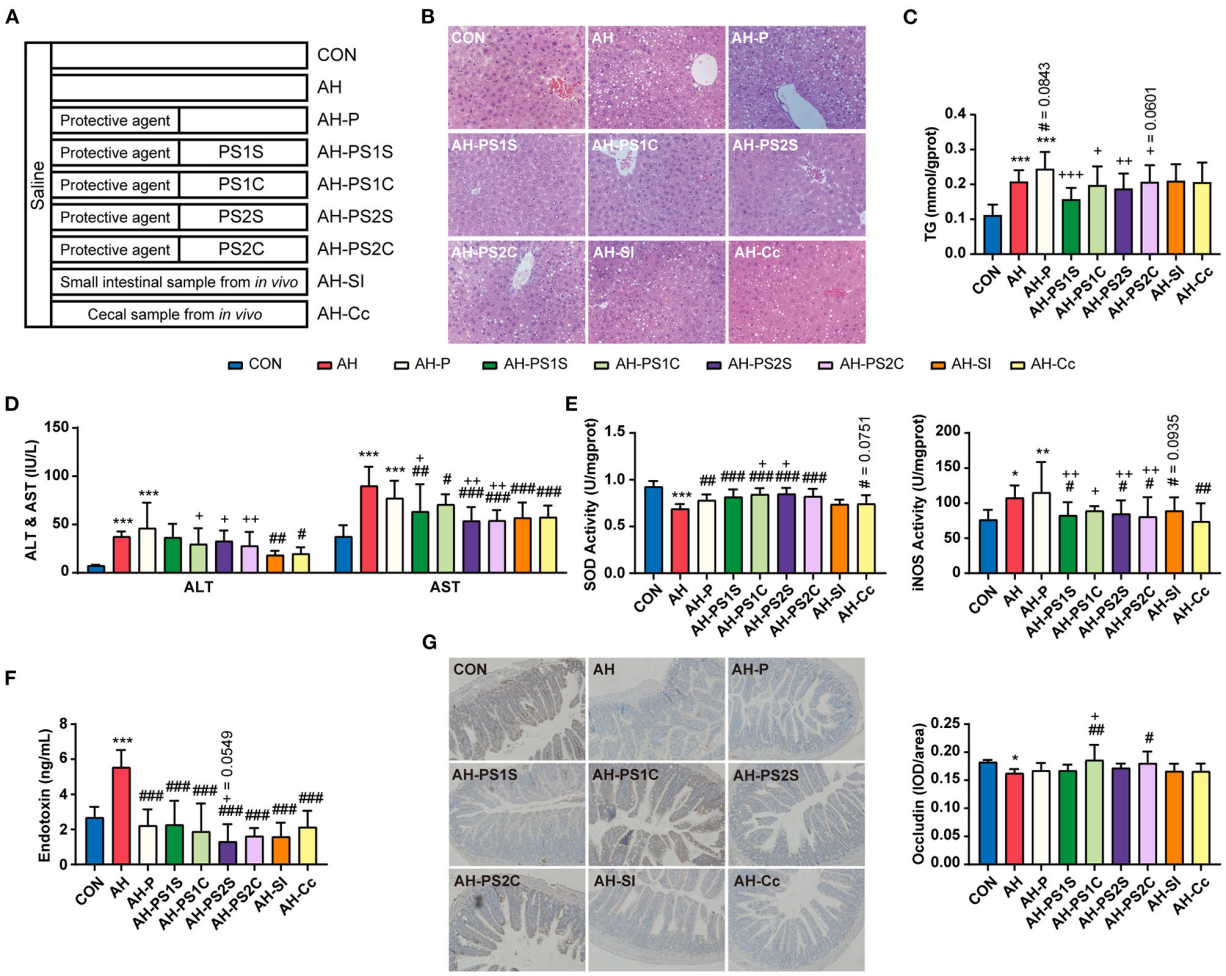
Effects of SCE-altered *in vivo*/*vitro* IM and its metabolites on liver and small intestine in AH mice. **(A)** Administration details in each group (*n* = 12 mice/group). Mice in groups beginning with “C” were fed with Lieber DeCarli control liquid diet, and mice in groups beginning with “AH” were fed with Lieber DeCarli ethanol liquid diet. **(B)** Hepatic H&E staining (magnification: 400 ×). **(C)** Content of triglyceride in the liver. **(D)** ALT and AST in serum. **(E)** SOD and iNOS activities in the liver. **(F)** Serum LPS level. **(G)** Immunocytochemistry for occludin in the ileum (magnification: 400 ×). Results were shown as the mean ± SD. *p < 0.05, **p < 0.01, ***p < 0.001 compared with CON group, #*p* < 0.05, ##*p* < 0.01, ###*p* < 0.001 compared with AH group, and +*p* < 0.05, ++*p* < 0.01, +++*p* < 0.001 compared with AH-P group by ANOVA one-way statistical analysis.

To determine whether SCE-altered IM and its metabolites modulate disease state, round-two animal experiment was carried out (Figure 4, Supplementary Figure 8). The effects of protective agent, which was used in *in vitro* samples to preserve as many live bacteria as possible, on the body were explored in this experiment to serve as a control for *in vitro* samples. Although the protective agent had beneficial effects on some indicators, it would not interfere with the exploration of the effects of *in vitro* samples. Alcohol-induced liver injury (serum ALT and AST, and liver index), enhancement of hepatic iNOS activity, and increased expression of *Cyp2e1* and inflammatory cytokines in the liver were inhibited to varying degrees in all treatment groups. The changes of hepatic triglyceride (TG) levels were consistent with the changes of fat vacuoles in hepatic H&E staining. AH-PS1S and AH-PS2S improved liver steatosis, and showed better anti-inflammatory effects than the other groups. AH-PS2S had an additional reduction in serum LPS levels, and the *in vivo* samples significantly decreased it. AH-PS2S had an additional increase in the expression of *Bmi1*. All treatment groups showed an increase in villus length and crypt depth. Additionally, according to the immunohistochemical test of occludin in the small intestine, we observed that a significant increase in occludin only appeared in the AH-PS1C and AH-PS2C groups compared with the AH group. These results indicated that the IM and its metabolites altered by SCE could improve the disease state to some extent.

### Effects of SCE-Altered *in vivo*/*vitro* IM and Its Metabolites on IM and SCFAs, and Their Correlation With Host Parameters

**Figure 5:**
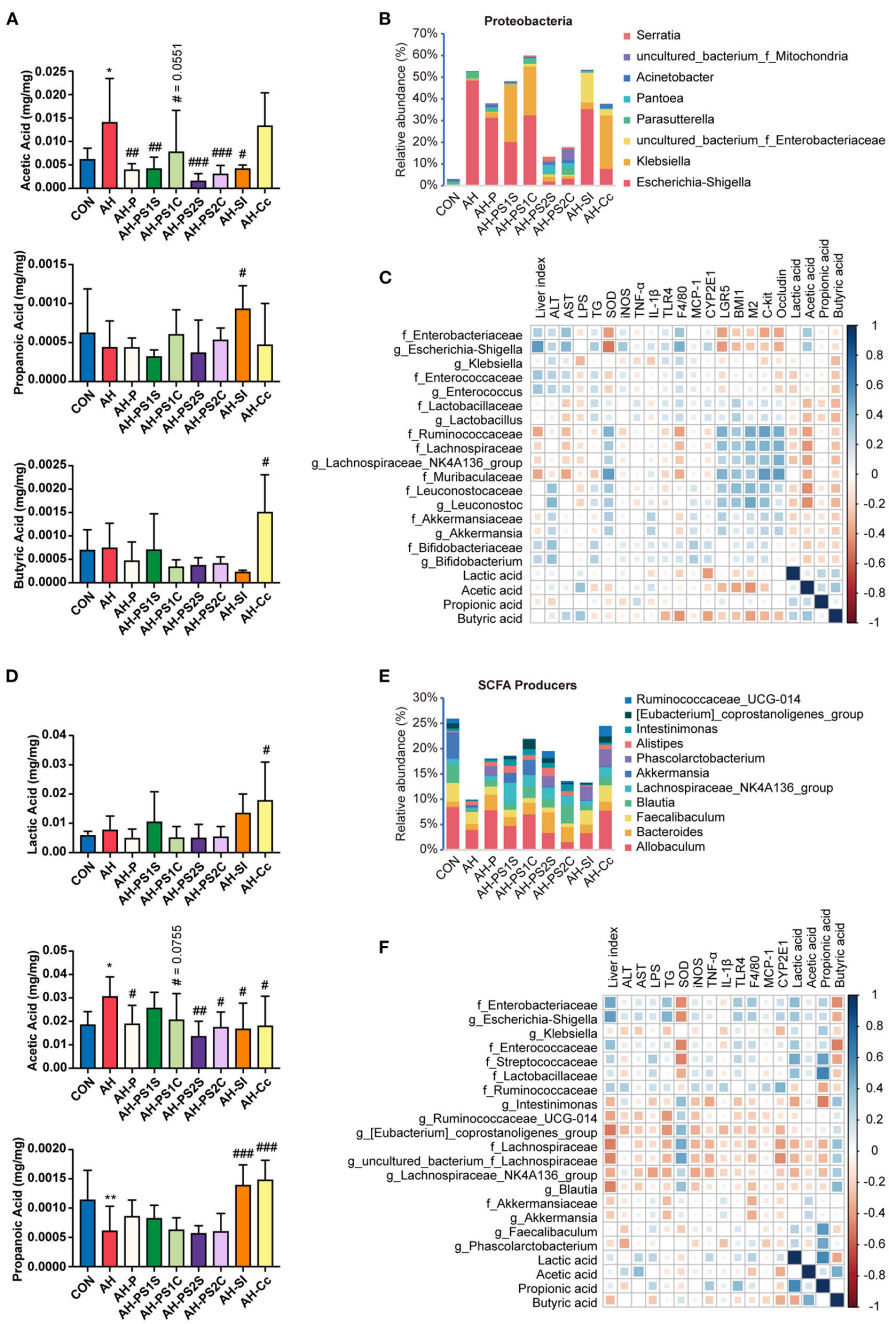
Effects of SCE-altered *in vivo*/*vitro* IM and its metabolites on IM and SCFAs in AH mice, and correlation between IM and host parameters. **(A)** SCFA content and **(B)** bacterial composition at the genus level in Proteobacteria in small intestine. **(C)** Spearman correlation of small intestinal microbiota and host parameters. **(D)** SCFA content and **(E)** relative abundance of SCFA producers in cecum. **(F)** Spearman correlation of cecal microbiota and host parameters. Results **(A,D)** were shown as the mean ± SD. *p < 0.05, **p < 0.01 compared with CON group, and #*p* < 0.05, ##*p* < 0.01, ###*p* < 0.001 compared with AH group by ANOVA one-way statistical analysis.

The overall effect of SCE administration on the microbial composition of the small intestine and cecum and the content of SCFAs in mice were evaluated (Figure 5, Supplementary Figures 9, 10). In the small intestine (Figures 5A,B, Supplementary Figure 9), the abundance of *Escherichia-Shigella* was markedly enriched in the AH group, but was diminished in all treatment groups, with the greatest reduction in the AH-PS2S, AH-PS2C, and AH-Cc groups. *Enterococcus* was found in an increased proportion in the AH-PS1S and AH-SI groups. The abundance of *Klebsiella* was enriched in the AH-PS1S, AH-PS1C, and AH-Cc groups. In contrast to the AH group, groups given *in vitro* SCE-altered IM samples showed varying degrees of increase in the abundance of some SCFA producers, Ruminococcaceae, Lachnospiraceae, *Lactobacillus, Akkermansia*, and *Bifidobacterium*. The AH-SI and AH-Cc groups showed an increase in the abundance of *Lactobacillus*. Lactic, propionic, and butyric acid contents were reduced, whereas the content of acetic acid increased in the AH group. Except for the AH-Cc group, the acetic acid level in the other treatment groups was significantly reduced. The content of propionic acid in the AH-SI group increased, and that of lactic acid and butyric acid in the AH-Cc group increased. In the cecum (Figures 5D,E, Supplementary Figure 10), the treatment groups showed different degrees of increase in the abundance of Lachnospiraceae, *Blautia, Lachnospiraceae_NK4A136_group, Akkermansia, Oscillibacter*, and *Bacteroides* compared to the abundance in the AH group. The *in vitro* SCE-altered IM samples had no significant effect on SCFA content. Both AH-SI and AH-Cc resulted in an increase in propionic acid and a decrease in acetic acid. AH-Cc was enriched with lactic acid. Additionally, the AH-Cc group showed an increase in the abundance of total SCFA producers in both the small intestine and cecum.

The potential correlation between changes in microbiota, metabolites in different intestinal segments, and liver injury, intestinal barrier functions in the host was assessed using Spearman correlation analysis. In the small intestine (Figure 5C), *Escherichia-Shigella* and *Enterococcus* were associated with aggravating the progression of AH and positively correlated with acetic acid. Lactobacillaceae, Bifidobacteriaceae, Ruminococcaceae, Lachnospiraceae, Muribaculaceae, Leuconostocaceae, and Akkermansiaceae were related to improved AH. We also found negative correlations between *Klebsiella* and serum LPS and the expression of some liver inflammatory cytokines, which were different from the results of the round-one animal experiment. In the cecum (Figure 5F), *Escherichia-Shigella*, Enterococcaceae, and Streptococcaceae were correlated with disease aggravation. *Intestinimonas, Ruminococcaceae_UCG_014, [Eubacterium]_coprostanoligenes_group, Lachnospiraceae_ NK4A136_group, Blautia*, and *Akkermansia*, which are SCFA producers, were found to alleviate AH. *Klebsiella* was negatively correlated with liver injury and the expression of some inflammatory cytokines. Moreover, butyric acid was negatively correlated with bacteria related to aggravating AH and positively correlated with some SCFA producers.

### Effects of Strains on Alcohol-Induced Liver Injury, Lipid Accumulation, Inflammation, and Intestinal Functions

**Figure 6:**
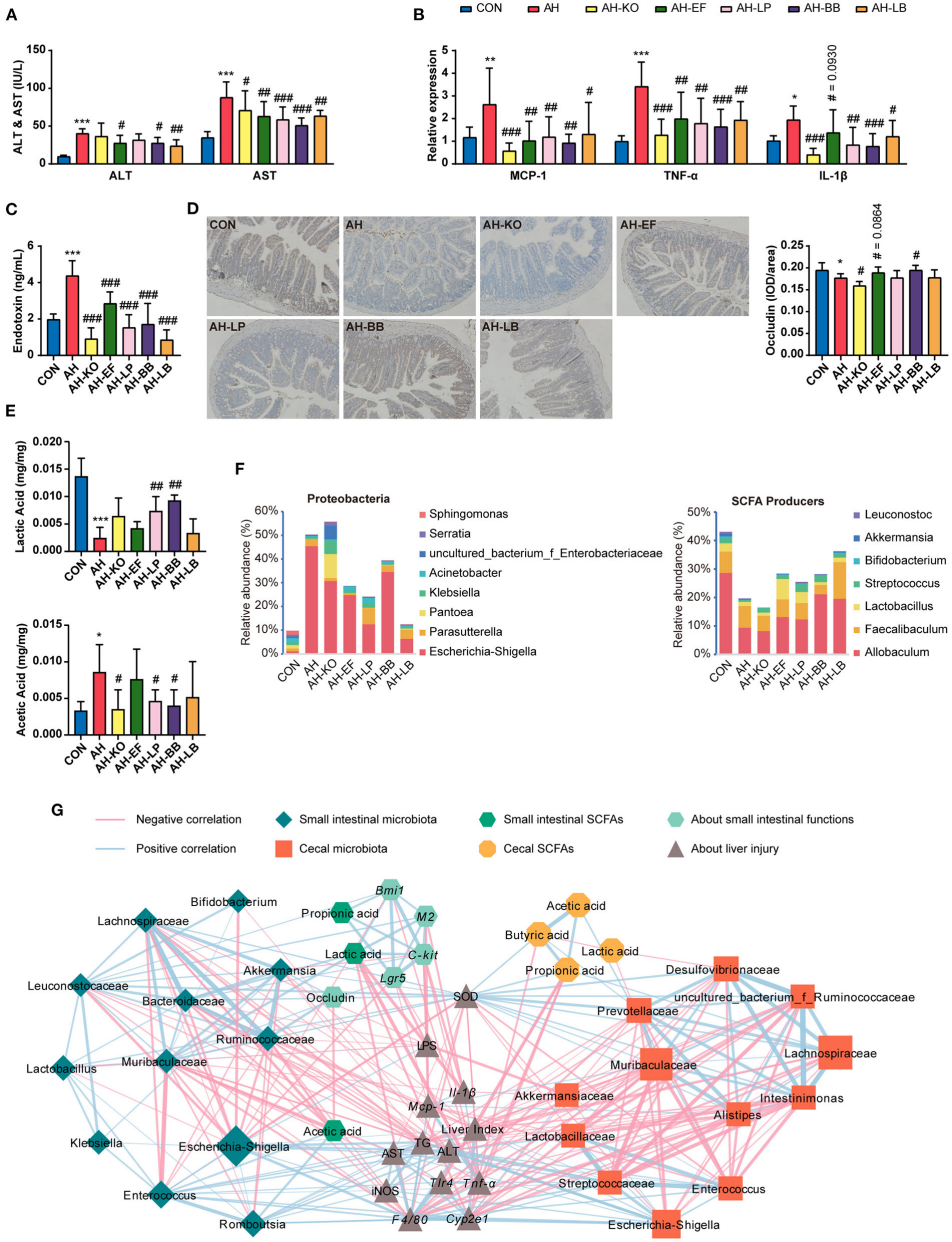
Effects of strains on liver, small intestine, IM and SCFAs in AH mice, and correlation between IM and host parameters. **(A)** ALT and AST in serum. **(B)** Hepatic mRNA expression of *Mcp-1, Tnf-*α, and *Il-1*β. **(C)** Serum LPS level. **(D)** Immunocytochemistry for occludin in the ileum (magnification: 400 ×). **(E)** SCFA content in the small intestine. **(F)** Bacterial composition at the genus level in Proteobacteria, and relative abundance of SCFA producers in the small intestine. **(G)** Spearman correlation of small intestinal and cecal microbiota and host parameters (*p* < 0.05). Results **(A–E)** were shown as the mean ± SD. *p < 0.05, **p < 0.01, ***p < 0.001 compared with CON group, and #*p* < 0.05, ##*p* < 0.01, ###*p* < 0.001 compared with AH group by ANOVA one-way statistical analysis.

The above experiments suggested that SCE improved the liver status of AH with the assistance of the modulated IM and its metabolites. Mice were administered with strains to explore the role of SCFA producers in SCE strengthening the intestinal barrier and the possible microbial targets of SCE to improve AH (Figure 6, Supplementary Figure 11). One strain of *Lactobacillus* (*L. plantarum*), which was related to improving AH with consistent results in the two rounds of experiments, one strain each of *Klebsiella* and *Enterococcus* (*K. oxytoca* and *E. faecalis*, which were detected in 16S rRNA sequencing in round-one animal experiment), which had different trends in the above experiments, and one strain of *Bifidobacterium* (*B. breve*), which was enriched by SCE *in vivo* and *in vitro*, were selected.

The changes of hepatic TG levels were consistent with the changes of fat vacuoles in hepatic H&E staining. Liver lipid accumulation in all treatment groups was not relieved (Supplementary Figures 11A,B). Compared with the AH group, all treatment groups showed improved liver status and decreased serum LPS levels. AH-KO showed the lowest expression of *Cyp2e1* and inflammatory cytokines in the liver. AH-KO and AH-LB showed lower serum LPS levels than the other groups (Figures 6A–C, Supplementary Figures 11C,D). Two probiotics and AH-EF improved intestinal barrier to different degrees, while the content of occludin decreased in the AH-KO group (Figure 6D, Supplementary Figures 11E,F).

*Klebsiella oxytoca* is an opportunistic pathogen of antibiotic-associated hemorrhagic colitis (33); hence, we investigated the impact of these strains on the colon (Supplementary Figure 12). The results of gene expression and H&E staining showed that these strains did not have an additional burden on the colon.

In this experiment, treatment groups improved the damage of alcohol to the liver and intestine to varying degrees. AH-KO had excellent performance in reducing oxidative stress, inflammation, and serum LPS levels and did not appear to cause symptoms of hemorrhagic colitis.

### Effects of Strains on IM and SCFAs, and Their Correlation With Host Parameters

The overall effect of the administration of strains on the microbial composition of the small intestine and cecum and the content of SCFAs in mice were evaluated (Figures 6E,F, Supplementary Figures 13, 14). In the small intestine, the proportions of Ruminococcaceae, Lachnospiraceae, and *Bifidobacterium* increased in AH-LB; proportions of Moraxellaceae, *Enterococcus*, and *Lactobacillus* were enriched in AH-EF and AH-LP; *Escherichia-Shigella* was of low abundance in AH-KO, AH-EF, AH-LP, and AH-LB. In the AH-KO group, *Enterococcus* increased, and *Lactococcus* decreased. The abundance of Muribaculaceae increased in AH-BB. The proportion of SCFA producers was decreased in the AH group, while it increased in AH-EF, AH-LP, AH-BB, and AH-LB. The lactic acid content increased in the AH-LP and AH-BB groups, and the acetic acid content decreased in all treatment groups. In the cecum, Erysipelotrichaceae increased in AH-LB group; Lactobacillaceae increased in all treatment groups except for AH-LB; *Escherichia-Shigella* decreased in AH-EF, AH-LP, and AH-LB groups; *Parabacteroides* increased in AH-LP group; *Klebsiella* and *Lachnospiraceae_NK4A136_group* increased in the AH-KO group; *Enterococcus* increased in the AH-KO and AH-EF groups. The total proportion of SCFA producers increased in the AH-LB group, while the acetic acid content decreased in the AH-EF and AH-BB groups and greatly decreased in the AH-LB group; propionic acid increased significantly in the AH-KO and AH-LP groups; butyric acid increased significantly in the AH-LP group.

The Spearman correlation analysis results were similar to the previous analyses (Figure 6G). In the small intestine, *Escherichia-Shigella, Enterococcus*, and *Romboutsia* were positively associated with each other and positively correlated with liver injury, inflammation, and intestinal acetic acid. *Escherichia-Shigella* was also negatively correlated with hepatic SOD activity and intestinal barrier and lactic acid. Ruminococcaceae, Lachnospiraceae, Muribaculaceae, Leuconostocaceae, Bacteroidaceae, *Lactobacillus, Bifidobacterium*, and *Akkermansia* were positively associated, and correlated with improved AH. Lactic acid was associated with improved disease symptoms. In the cecum, *Escherichia-Shigella*, Enterococcaceae, and Streptococcaceae were positively associated with each other and correlated with aggravating AH, and Lachnospiraceae, Muribaculaceae, Desulfovibrionaceae, Preotellaceae, *Insominimonas*, and *Alistipes* were positively associated with each other and correlated with improved AH. Propionic acid and butyric acid were associated with improved disease status. Additionally, in both the small intestine and cecum, Lactobacillaceae (*Lactobacillus*) was positively associated with *Enterococcus*.

### Effects of Two Lignans in SCE on AH

Since lignans are the most important components of *S. chinensis* fruit, among which schisandrol A and schisandrin B are representative ones, mice were administered with either schisandrol A or schisandrin B in order to investigate whether they play a role in improving AH. The results of hepatic H&E staining, serum ALT, AST and LPS, hepatic oxidative stress enzymes, and inflammatory cytokine expression showed that neither schisandrol A nor schisandrin B improved AH. Additionally, schisandrol A increased serum ALT and AST levels, and schisandrin B increased the expression of inflammatory cytokines in the liver (Supplementary Figure 15).

## Discussion

Substantial reports suggested that changes in gut microbiota are an important part of the pathogenesis of AH. *Schisandra chinensis* has been shown the potential to ameliorate alcohol- induced liver injury, however, the relationship between its therapeutic effects and gut microbiota is still unclear. This study was based on the AH simulated by a chronic-plus-binge ethanol feeding model. Firstly, it was determined in a pre-experiment that SCE ameliorated AH non-dose-dependently. Then, under the most effective dose, the possibility of SCE to prevent liver damage and destruction of the intestinal barrier caused by alcohol intake and regulate IM was investigated. Finally, the role played by IM and its metabolites in the prevention of AH by SCE was explored, and the possible microbial targets were validated.

In this study, in addition to suppressing hepatic lipid accumulation, SCE effectively inhibited liver inflammation. Oxidative stress is an important factor in the development of alcohol-induced liver inflammation, and inhibition of oxidative stress has been shown to be a mechanism by which SCE improves ALD (11). Our results on anti-oxidant, including SOD and *Cyp2e1*, were consistent with previous reports. In addition, we also noticed that SCE attenuated nitrosative stress by inhibiting iNOS activity, thereby playing a beneficial role in liver inflammation.

The increased bacterial product, LPS, in the portal vein caused by disruption of intestinal barrier integrity also contributes to the development of inflammation in AH (3). The significant reduction in serum LPS level by SCE administration can be linked to the multiple enhancement of intestinal barrier functions, including intestinal stem cells, tight junction, and intestinal peristalsis. In addition to the intestinal barrier of the host, the IM and metabolites have profound effects on intestinal function (34). Many studies have shown an increase in the proportion of Proteobacteria in AH, and we observed consistent results. SCE reduced the proportion of Proteobacteria, which is the main source of LPS. *Escherichia-Shigella* inhibited by SCE was associated with aggravated AH, which was related to its disruption of the intestinal barrier. SCFA-producing bacteria and lactic acid bacteria (LAB), especially *Lactobacillus, Bifidobacterium*, and Lachnospiraceae, were enriched in SCE treatment group and altered intestinal SCFAs and lactate levels, which can contribute to the improvement of intestinal functions by SCE. SCFAs, metabolized by gut bacteria from indigestible carbohydrates, help improve the intestinal barrier, protect against enterotoxins, and regulate the microbiome (35). Lactate supports the integrity of the intestinal mucosa, modulates the IM, and can be metabolized to butyrate, propionate, and succinate (36). In addition, the results of the animal experiment and *in vitro* bacteria growth experiments consistently showed that SCE selectively inhibited microbial growth. Gut microbiota composition is well-established to change after alcohol intake, and the alterations worsen with advancing disease (37). Therefore, we hypothesized that SCE could exert therapeutic effects by modulating the dysregulated gut microbiota and associated metabolites.

To verify the above hypothesis, we collected SCE-altered intestinal samples obtained from *in vivo* and *in vitro* experiments and gavaged them to mice for intestinal microbiota transplantation. Although the compositions of *in vivo* and *in vitro* IM modulated by SCE changed differ, which may due to the presence or absence of a strictly anaerobic environment and organismal feedback, their effects on the body were similar in that they both improved liver status, including inflammation and oxidative/nitrosative stress, but had a weak effect on intestinal barrier function. Intestinal microbiota transplantation also reduced serum LPS level and the proportion of *Escherichia-Shigella*, the main pathogenic bacteria in Proteobacteria, and increased the proportion of *Lactobacillus* in the small intestine and some SCFA-producers in the cecum. These results suggested that the improvement of liver status in AH by SCE is closely associated with IM. However, due to the weak effects of intestinal microbiota transplantation on intestinal barrier function, the relationship between IM and the intestinal health of AH mice is puzzling.

According to the process of alcohol digestion and absorption, the small intestine is exposed to more alcohol (32), while the cecum is a common site for detecting intestinal microbes in mice. Therefore, we investigated the IM composition and SCFAs levels in the small intestine and cecum. We noticed that alcohol intake led to greater changes in the composition of microorganisms in the small intestine than in the cecum, whereas the effects of intestinal microbiota transplantation using samples from the small intestine and the cecum on AH were similar. Despite the presence of a greater proportion of SCFA producers in the small intestine than in the cecum, intestinal microbiota transplantation of small intestinal samples did not result in recipient mice with more intestinal SCFA producers. These suggested that small intestinal samples are more sensitive for disease detection and diagnosis, without any advantage in the selection of transplanted samples. Microorganisms were not simply colonized when transplanted to a host, and the crosstalk involved made the potential link between IM and host status not as strong. Transplantation of *in vitro* samples obtained by maintaining an anaerobic environment with CO_2_ and N_2_, which resulted in a remarkably low proportion of Proteobacteria in the small intestine and a low proportion of *Escherichia-Shigella* in the cecum, appeared to cause a lower serum LPS level, but with no additional beneficial effects on intestinal barrier. Therefore, we hypothesized that SCE improved the intestinal barrier function in AH mice by increasing the proportion of SCFA producers.

To explore the possible microbial targets of SCE to improve AH and the role of SCFA producers, several strains were selected for administration to AH mice. *Enterococcus faecalis, Lactobacillus plantarum*, and *Bifidobacterium breve* resulted in increased small intestinal SCFA producers and varying degrees of enhancement of the intestinal barrier. Correlation network also demonstrated a potential positive relationship among SCFA producers, as well as an association with the improvement of liver inflammation. These results suggested that the enhancement of intestinal barrier function in AH by SCE is closely associated with SCFA producers.

*Shigella* is a gram-negative intestinal pathogen with a very low infectious dose and can cause intestinal inflammation and loss of intestinal barrier function (38). Both *in vivo* and *in vitro* we observed the inhibition of SCE on *Escherichia-Shigella*/*Shigella flexneri* growth. Hence, *Escherichia-Shigella* is a microbial target of SCE. *Bifidobacterium* and *Lactobacillus* strains are the most widely used probiotics in functional foods and dietary supplements (39). Indeed administration of different *Lactobacillus, Bifidobacterium*, or their supernatants can improve alcohol-induced liver damage, intestinal barrier function, and IM (40, 41). Consistent results were obtained in this study. *Enterococcus* is a LAB that can produce natural lactic acid and bacteriocin (42) and influence the intestinal microbial community by fermenting carbohydrates and amino acids to provide substrates for the growth of other species and form secondary metabolites, thus improving the richness of specific symbiotic bacteria (43), however, the cytolysin produced by *Enterococcus* can aggravate alcohol-induced liver disease (44). In this study, we observed a mutual promotion between *Lactobacillus* and *Enterococcus*. *Enterococcus faecalis* had a beneficial effect on AH, which probably due to the regulation of the IM and SCFAs. Moreover, the conflicting effects of *E. faecalis* on AH is also attributed to different isolated and investigated strains (45). Interestingly, *Klebsiella oxytoca* improved liver injury and regulation of the IM and SCFAs. Currently, most research on *K. oxytoca* focuses on the increase in the proportion of *K. oxytoca* in diseases such as colitis, meningitis, and pneumonia. Tilivalline and tilimycin secreted by *K. oxytoca* are the main causes of antibiotic-associated hemorrhagic colitis as they can increase cell apoptosis and reduce the expression of tight junction proteins to induce intestinal barrier damage (46). We also observed adverse effects of *K. oxytoca* on intestinal tight junction proteins. However, *Klebsiella oxytoca* produces the byproducts, such as acetic acid, lactic acid, succinic acid and formic acid (47), which can be of indirect beneficial effects on other aspects of the intestinal barrier or on the liver. Meanwhile, the improved liver inflammation caused by *K. oxytoca* presumably resulted from decreased LPS leakage from the intestine and its regulation of *Cyp2e1* expression. However, the specific mechanism by which *K. oxytoca* leads to a substantial decrease in serum LPS levels and the expression of liver inflammatory cytokines requires further investigation. Therefore, *Escherichia-Shigella, Lactobacillus*, and *Bifidobacterium* are possible targets for SCE to regulate intestinal microorganisms to improve AH, while *E. faecalis* and *K. oxytoca* remain unclear.

Regulation of intestinal microbes has become a therapeutic direction for the treatment of AH/ALD in clinical trials. There are three main methods for regulating intestinal microbes: antibiotic administration, probiotic supplementation, and fecal microbiota transplantation (FMT) (48). Broad-spectrum antibiotics administered in combination [vancomycin, gentamycin, and meropenem (49)] or alone [paromomycin (50)], did not improve ALD. Rifaximin exhibited beneficial effects when used to treat hepatic encephalopathy (51), and was considered to have the potential to treat alcoholic liver cirrhosis (52). Additionally, FMT reportedly increases the survival rate of patients with AH (53). Intestinal microbiota transplantation using intestinal microorganisms with drug intervention in an *in vitro* simulation system provides new insights for FMT. Compared with intestinal microbiota transplantation and probiotic treatment, SCE administration provided a more comprehensive and stable therapeutic effect. Thus, the selective inhibition of intestinal microbes and potential as a source of prebiotics suggests that SCE can elicit advantages of both antibiotics and probiotics as a treatment target for regulating microbes. It is also worth exploring whether *S. chinensis* tea can be used as a regular drink to prevent AH/ALD.

Lignans are important constituents of *S. chinensis* fruit. However, schisanol A and schisanin B did not improve AH in this study. While most articles about schisanol A and schisanin B focus on their effects of improving diseases, some researchers have also focused on the hepatotoxicity of schisanin B (54). Although the disappointing results obtained with both lignans in this study may be related to the dose of administration, the comprehensive and effective improvement of AH by SCE presumably due to the combined effects of multiple bioactive substances.

**Figure 7:**
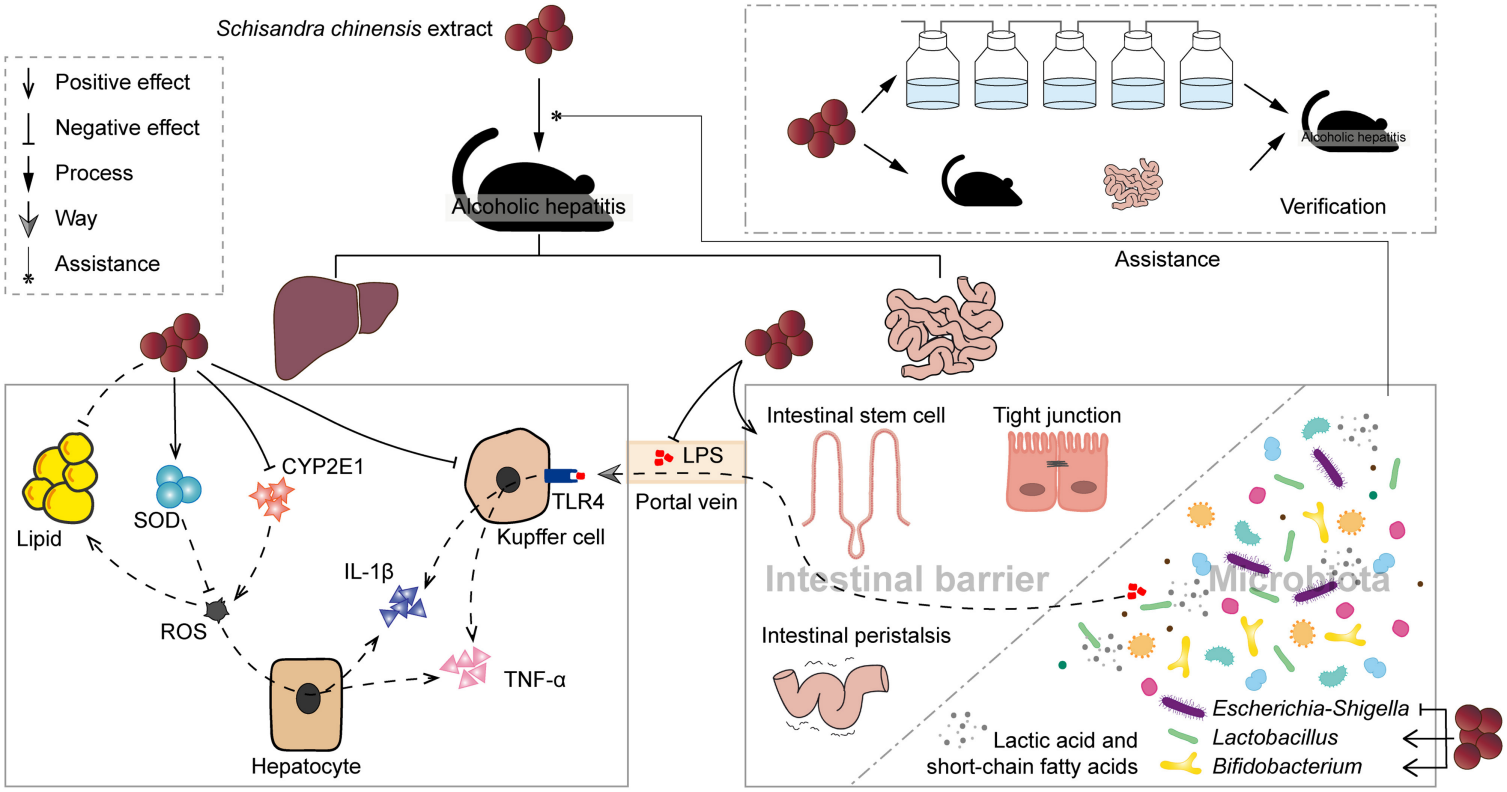
*Schisandra chinensis* extract improved alcoholic hepatitis in mice with the assistance of modulated intestinal microbiota.

Our study demonstrated the effectiveness of SCE on AH at the level of the liver, intestinal barrier, and the IM and its metabolites. Intestinal microbiota and their metabolites were closely associated with the improvement of SCE on AH, where the possible microbial targets include inhibition of *Escherichia-Shigella* and expansion of the proportion of SCFA/lactate-producing bacteria, such as *Lactobacillus* and *Bifidobacterium*, and SCFA/lactate-producing bacteria contributed to the strengthening of the intestinal barrier (Figure 7). Contrasting changes in small intestinal and cecal microbiota after alcohol consumption, changes in the microbial composition of the small intestine can be more sensitive to disease detection and diagnosis. *Schisandra chinensis* can be considered as a safe and effective dietary supplement for the prevention and improvement of AH. Our study will provide theoretical support for the clinical use of *S. chinensis* in the treatment of AH.

## Data Availability Statement

The data that support the findings of this study are openly available in Sequence Read Archive (SRA) at NCBI, reference numbers PRJNA574740, PRJNA748691, PRJNA748699, and PRJNA748718.

## Ethics Statement

The animal study was reviewed and approved by the Experimental Animal Welfare and Ethics Committee of School of Life Sciences, Jilin University.

## Author Contributions

J-YX: conceptualization, investigation, visualization, and writing—original draft preparation. Y-YC, J-XH, and XS: investigation. YC: resources and supervision. HX and QX: conceptualization, resources, supervision, and writing—review and editing. All authors contributed to the article and approved the submitted version.

## Funding

This work was supported by the Department of Science and Technology of Jilin Province [grant numbers 20200708072YY and 20210401122YY].

## Conflict of Interest

The authors declare that the research was conducted in the absence of any commercial or financial relationships that could be construed as a potential conflict of interest.

## Publisher's Note

All claims expressed in this article are solely those of the authors and do not necessarily represent those of their affiliated organizations, or those of the publisher, the editors and the reviewers. Any product that may be evaluated in this article, or claim that may be made by its manufacturer, is not guaranteed or endorsed by the publisher.

## Abbreviations

AH: Alcoholic hepatitis
SCE: Schisandra chinensis extract
IM: intestinal microbiota
SCFAs: short-chain fatty acids
ALD: alcoholic liver disease
LPS: lipopolysaccharide
TNF-α: tumor necrosis factor alpha
IL-1: interleukin-1
PBET: physiologically based extraction test
SHIME^®^: simulator of human intestinal microbial ecosystem
CMC: carboxymethyl cellulose sodium
NF-κB: nuclear factor kappa-B
ALT: alanine aminotransferase
AST: aspartate aminotransferase
SOD: superoxide dismutase
iNOS: inducible nitric oxide synthase
H&E: hematoxylin and eosin
TG: triglyceride
LAB: lactic acid bacteria
FMT: fecal microbiota transplantation.
