# Supplementary material for: Intestinal Microbiota Contributes to the Improvement of Alcoholic Hepatitis in Mice Treated With Schisandra chinensis Extract
Source: Front Nutr. 2022 Feb 18;9:822429. doi: 10.3389/fnut.2022.822429 (PMC8894320; doi:10.3389/fnut.2022.822429)
Supplement: Supplementary file 1 [file Data_Sheet_1.docx]

Intestinal microbiota contributes to the improvement of alcoholic hepatitis in mice treated with *Schisandra chinensis* extract

Jun-Yan Xiang,^1,2^ Yan-Yu Chi,^1,2^ Jin-Xin Han,^1,2^ Xinyu Shi,^1,2^ Yong Cai,^1,2,3,4*^ Hongyu Xiang^1,2,3,4*^ and Qiuhong Xie^1,2,3,4*^

^1^ Key Laboratory for Molecular Enzymology and Engineering of Ministry of Education, School of Life Sciences, Jilin University, Changchun 130012, People’s Republic of China

^2^ School of Life Sciences, Jilin University, Changchun 130012, People’s Republic of China

^3^ National Engineering Laboratory for AIDS Vaccine, School of Life Sciences, Jilin University, Changchun 130012, People’s Republic of China

^4^ Resources and Applied Microbiology Laboratory, Institute of Changbai Mountain Resource and Health, Jilin University, Fusong 134504, People’s Republic of China

* Corresponding authors:

Yong Cai; E-mail: caiyong62@jlu.edu.cn

Hongyu Xiang; E-mail: hyxiang@jlu.edu.cn

Qiuhong Xie; E-mail: qhxie@jlu.edu.cn

Tel & Fax: +86-431-85153832

ORCID ID: 0000-0002-3902-8436

**1. Results**

**1.1 Effects of SCE on metabolites**

In the metabolomic results of the small intestine and cecum determined using ^1^H NMR (Table S2–3), we observed that the main contents were ethanol and a variety of amino acids, and more amino acids were detected in the small intestine than in the cecum. Alcohol intake significantly increased the proportion of alcohol in the small intestine and cecum. In both the small intestine and the cecum, the total amino acid content in the AH group was reduced compared with that in the CON group, and increased after SCE administration. We observed an increase in the proportion of taurine, valine, leucine/isoleucine in the small intestine and an increase in the proportion of methionine in the cecum after administration. Overall, however, the metabolic changes in the model used in this study were not obvious.

**1.2 Effects of SCE on bacterial growth *in vitro***

To explore the influence of SCE on bacterial growth, we performed antibacterial experiments or bacterial growth experiments on 24 strains of bacteria (Tables S4–5). SCE had an obvious inhibitory effect on pathogens/conditioned pathogens/controversial bacteria, including *Shigella flexneri*, *Klebsiella oxytoca*, *and Enterococcus faecalis*. SCE had no significant effect on the *Lactobacillus* and *Bifidobacterium* species, but promoted *Lactobacillus royi* growth to some extent. These results indicate that SCE selectively inhibited bacterial growth.

**Figures**


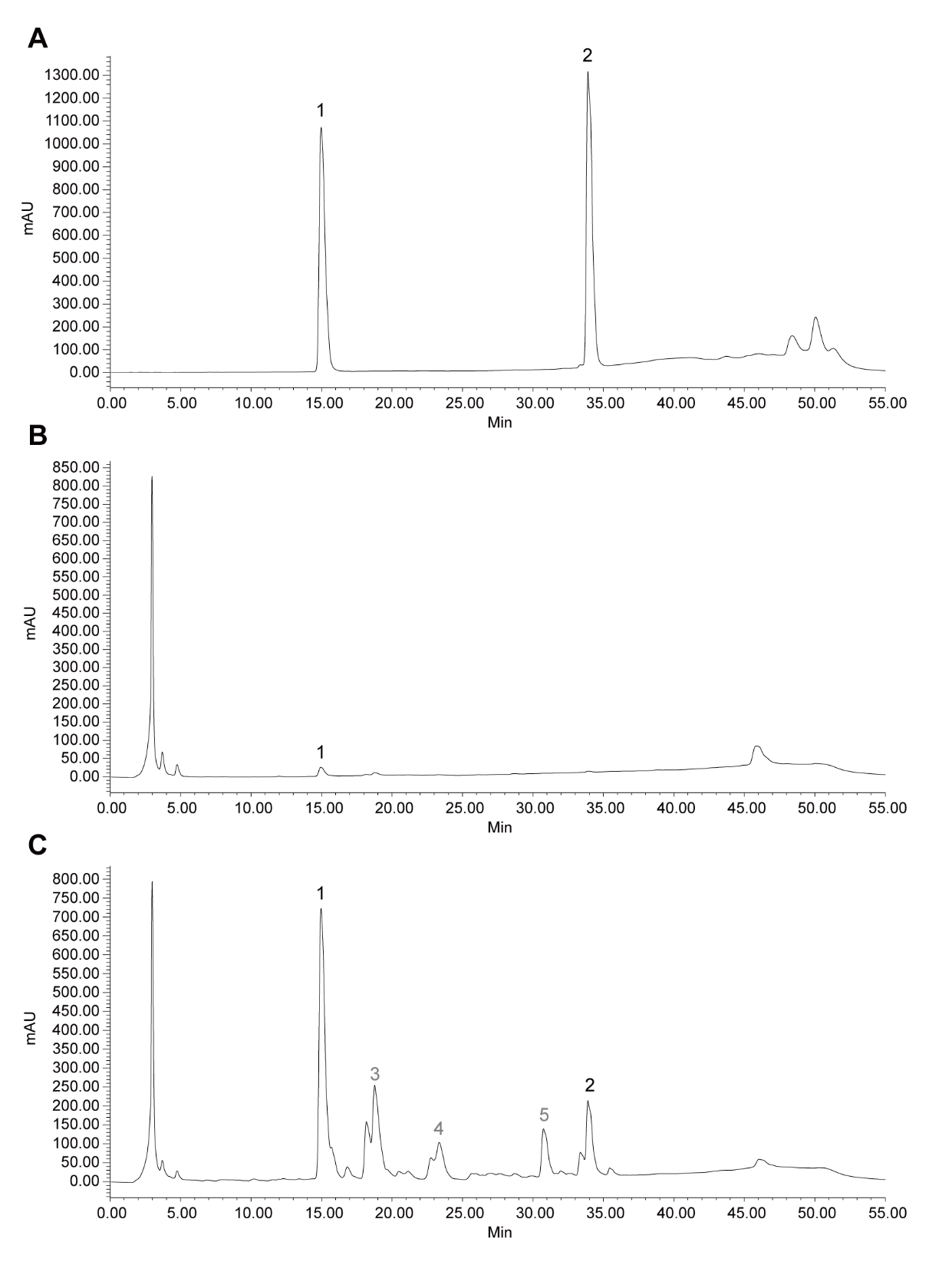


Fig. S1 Chromatograms of lignin reference substances, *S. chinensis* water extract and ethanol extract

(A) Mixed reference substances, (B) *S. chinensis* water extract, (C) *S. chinensis* ethanol extract. 1. Schisandrol A and 2. schisandrin B were reference substances determined in this experiment. 3. Schisandrol B, 4. schisantherin and 5. schisandrin A referred to the reference article [1].


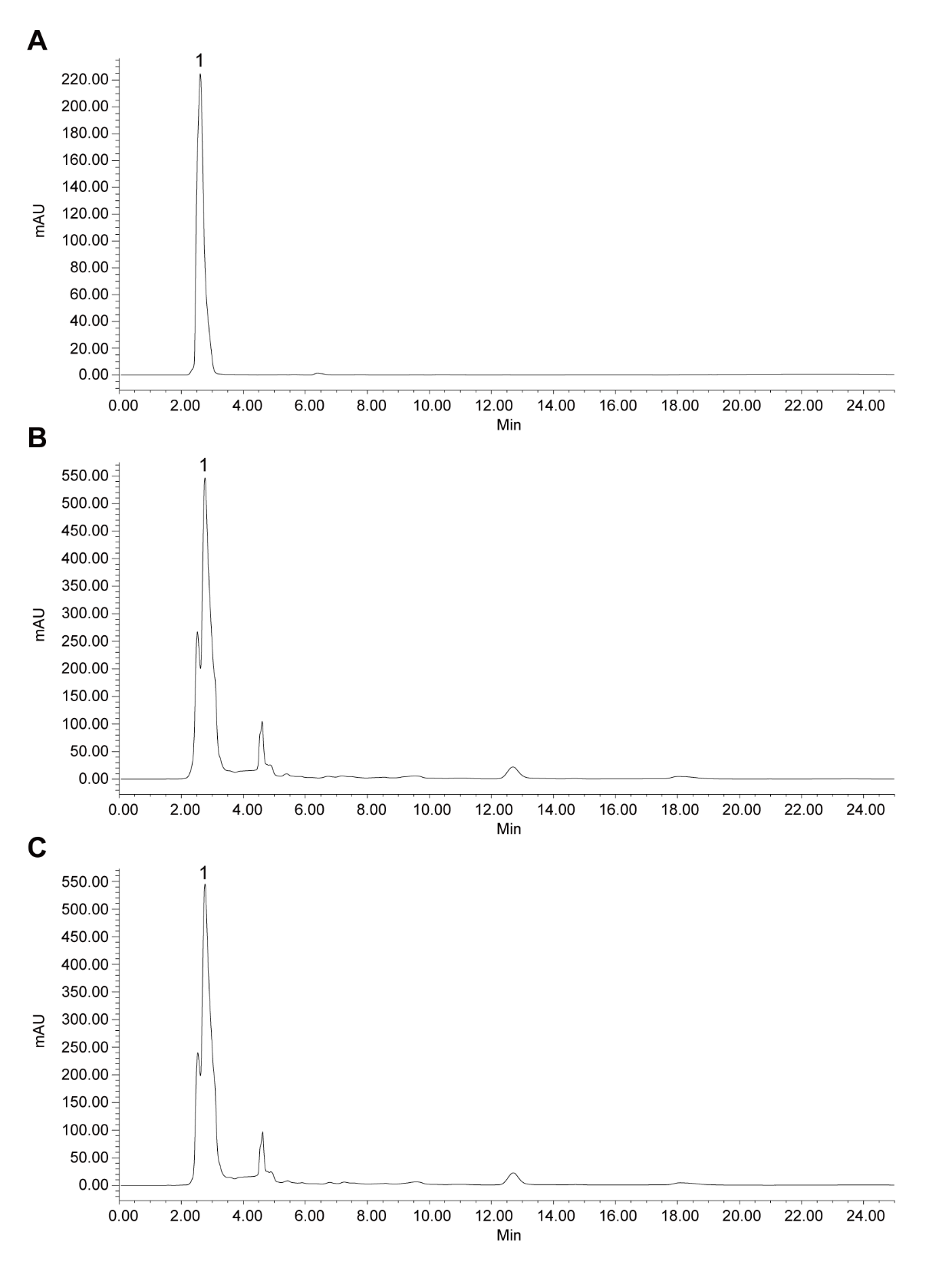


Fig. S2 Chromatograms of organic acid reference substances, *S. chinensis* water extract and ethanol extract

(A) Citric acid, (B) *S. chinensis* water extract, (C) *S. chinensis* ethanol extract. 1. Citric acid.


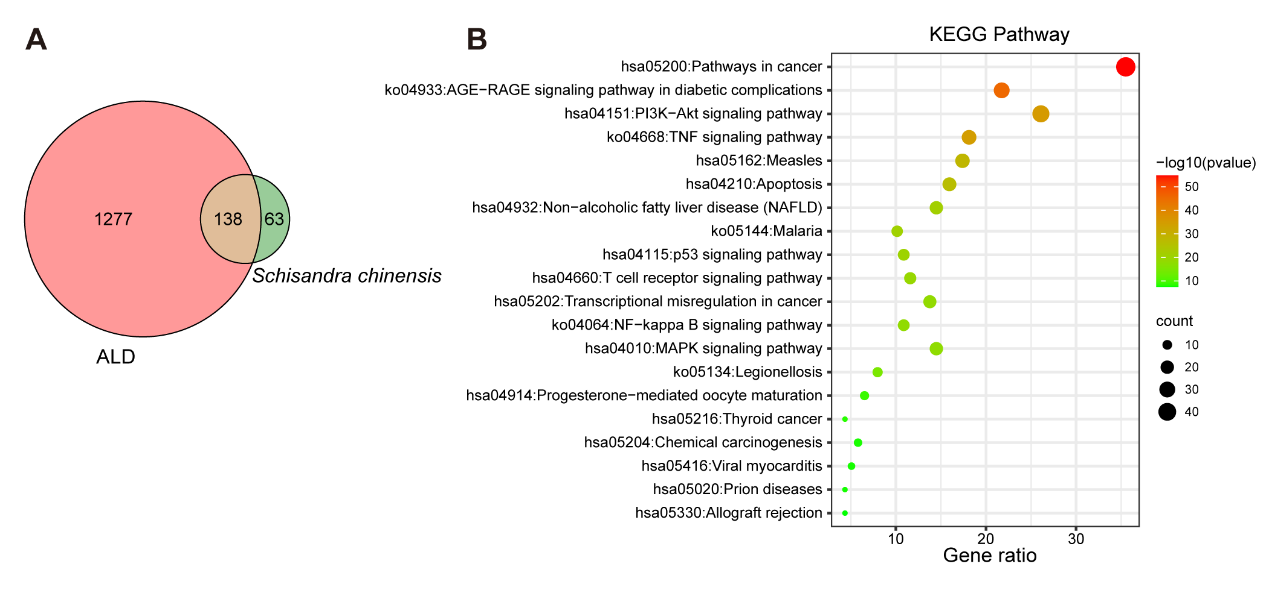


Fig. S3 Network pharmacology analysis of *S. chinensis* in the treatment of ALD

(A) Venn diagram for *S. chinensis* and ALD targets. The overlap targets mean the potential therapeutic gene for *S. chinensis* when treating ALD. (B) KEGG pathway enrichment.


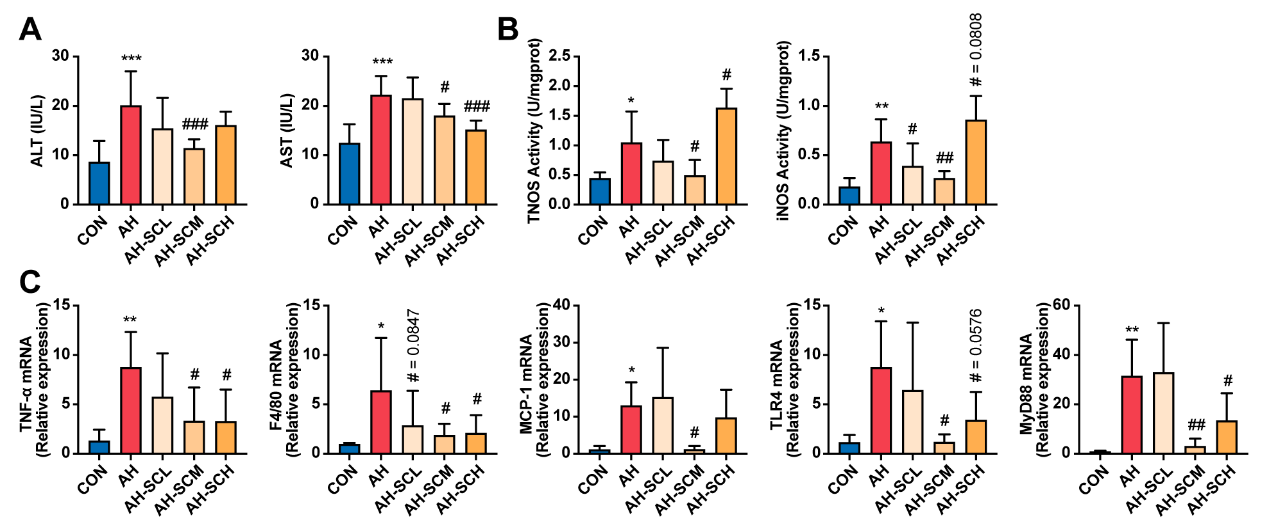


Fig. S4 Effects of different doses of SCE administration on AH

(A) ALT and AST in serum. (B) TNOS and iNOS activities in the liver. (C) Hepatic mRNA expression of TNF-α, F4/80, MCP-1, TLR4, and MyD88. Results were shown as the mean ± SD. * *p*<0.05, ** *p*<0.01, *** *p*<0.001 compared with CON group, and # *p*<0.05, ## *p*<0.01, ### *p*<0.001 compared with AH group by ANOVA one-way statistical analysis.


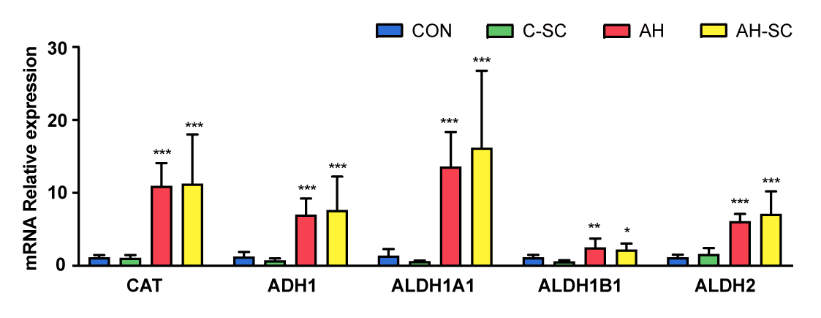


Fig. S5 Effect of SCE administration on the expression of genes involved in alcohol metabolism

mRNA expression of CAT, ADH1, ALDH1A1, ALDH1B1, ALDH2 in the liver. Results were shown as the mean ± SD. * *p*<0.05, *** *p*<0.001 compared with CON group by ANOVA one-way statistical analysis.


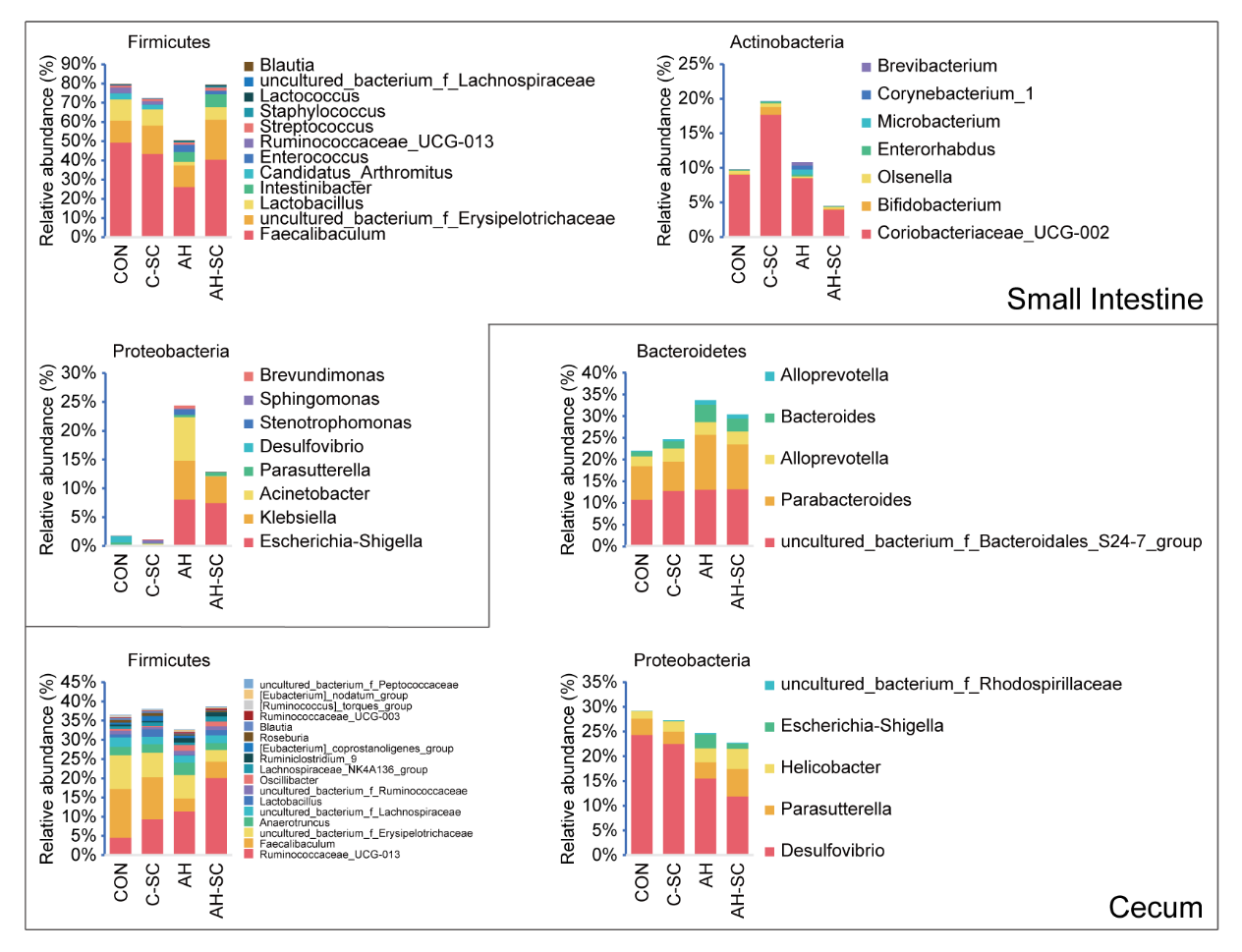


Fig. S6 Effect of SCE administration on the bacterial composition of the small intestine and the cecum

Bacterial composition at the genus level in Firmicutes, Actinobacteria and Proteobacteria in the small intestine, and bacterial composition at the genus level in Bacteroidetes, Firmicutes and Proteobacteria in the cecum.


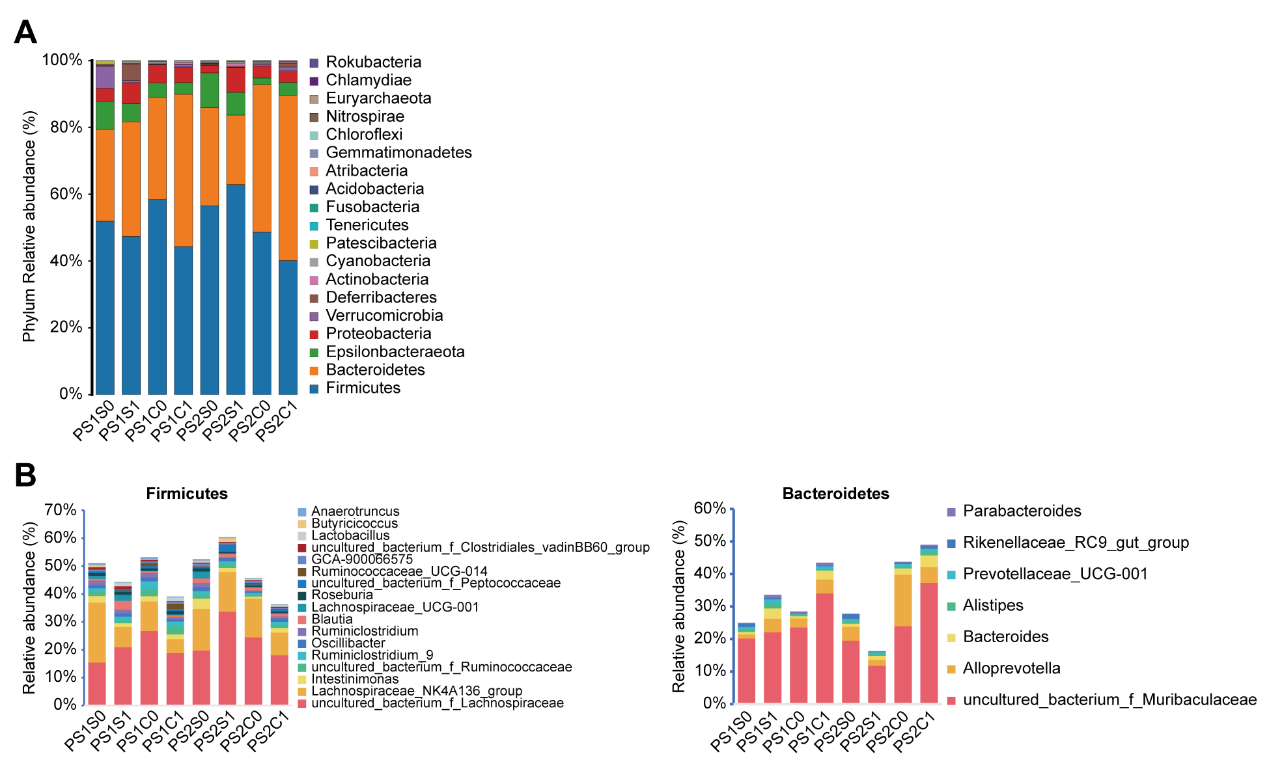


Fig. S7 Effects of SCE on intestinal microbe composition in an *in vitro* gastrointestinal simulation system

(A) Relative abundance of microbiota at the phylum level. (B) Bacterial composition at the genus level in Firmicutes and Bacteroidetes.


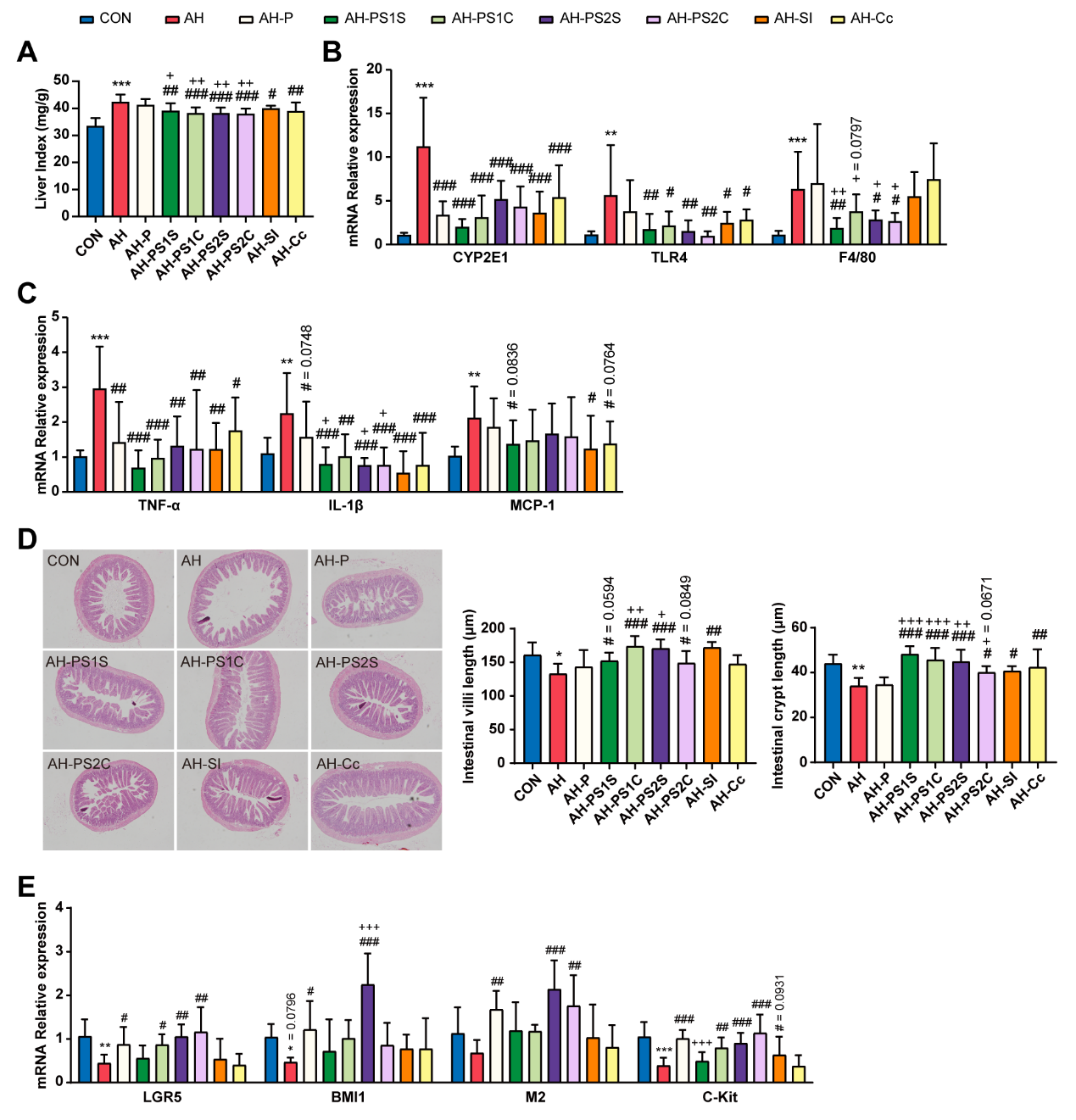


Fig. S8 Effect of intestinal microbiological samples administration on liver and small intestine

(A) Liver index. (B-C) Hepatic mRNA expression of CYP2E1, TLR4, F4/80, TNF-α, IL-1β and MCP-1. (D) Small intestinal H&E staining (magnification: 100×), and the length of villi and crypts. (E) Small intestinal mRNA expression of LGR5, BMI1, M2, and C-kit. Results were shown as the mean ± SD. * *p* < 0.05, ** *p* < 0.01, *** *p* < 0.001 compared with CON group, # *p* < 0.05, ## *p* < 0.01, ### *p* < 0.001 compared with AH group, and + *p* < 0.05, ++ *p* < 0.01, +++ *p* < 0.001 compared with AHP group by ANOVA one-way statistical analysis.


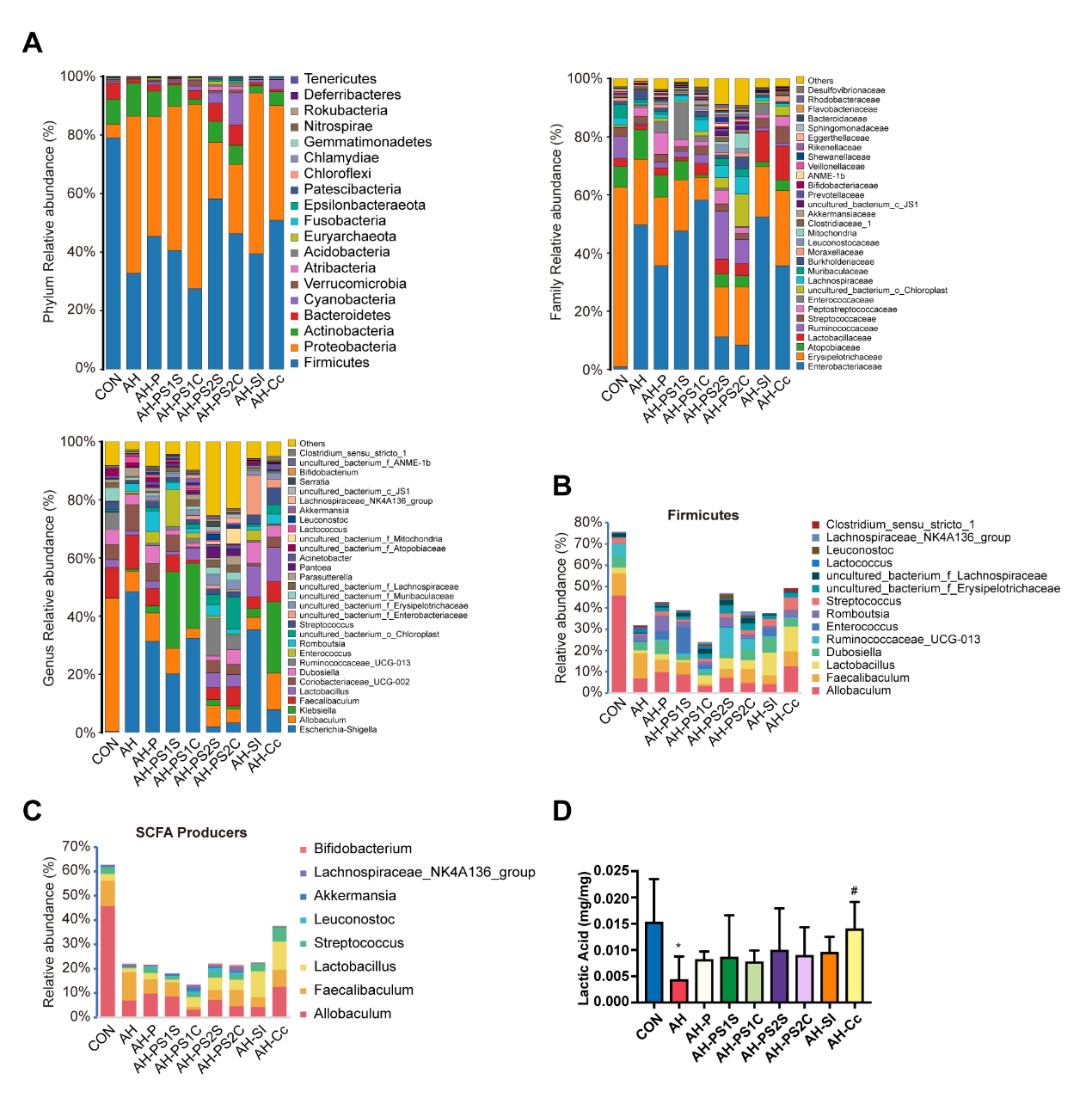


Fig. S9 Effect of intestinal microbiological samples administration on the bacterial composition and lactic acid of the small intestine

(A) Relative abundance of microbiota at phylum, family and genus levels. (B) Bacterial composition at the genus level in Firmicutes. (C) Relative abundance of SCFA producers. (D) Lactic acid level. Results (D) were shown as the mean ± SD. * *p* < 0.05 compared with CON group, and # *p* < 0.05 compared with AH group by ANOVA one-way statistical analysis.


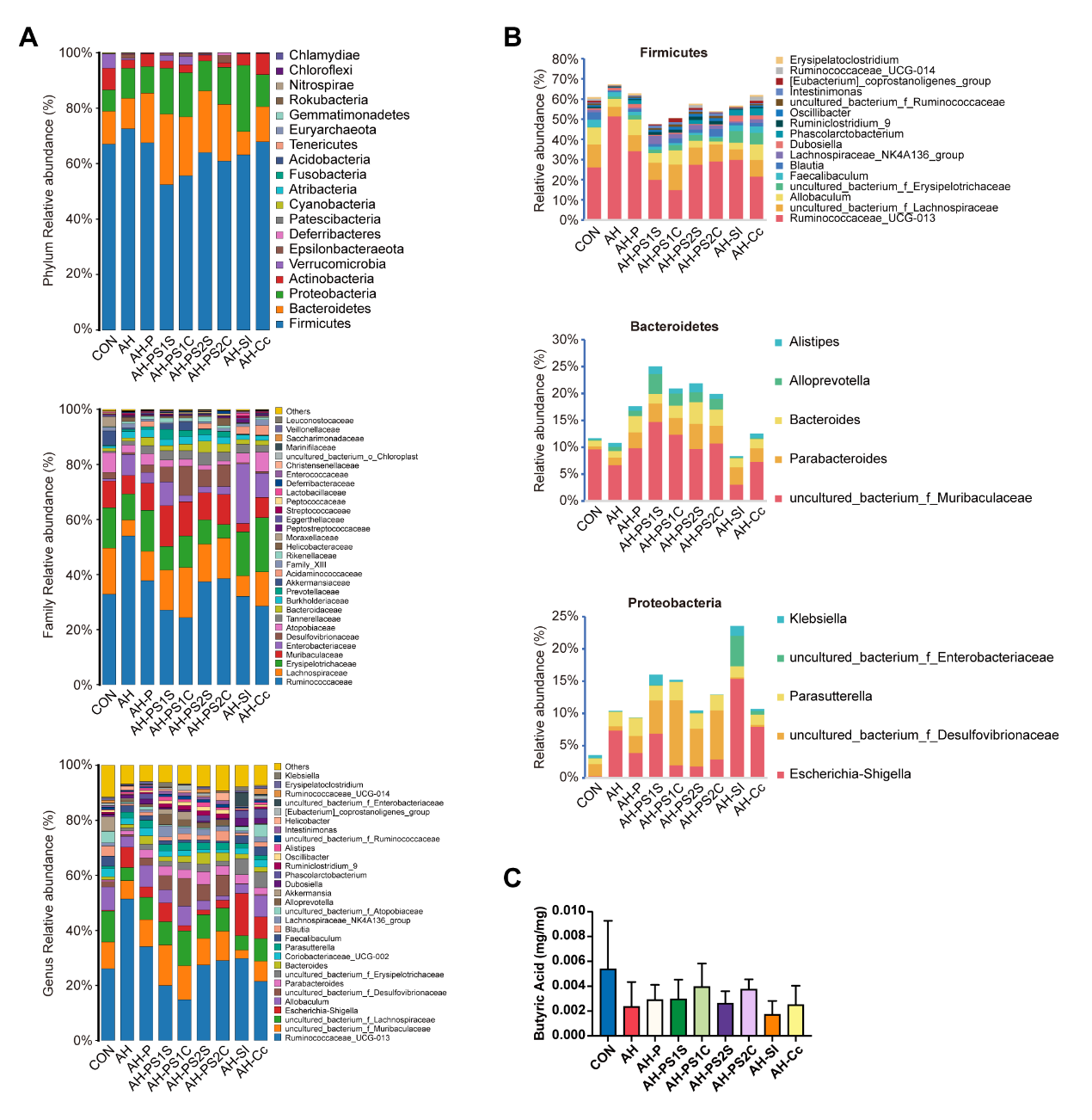


Fig. S10 Effect of intestinal microbiological samples administration on the bacterial composition and butyric acid of the cecum

(A) Relative abundance of microbiota at phylum, family and genus levels. (B) Bacterial composition at the genus level in Firmicutes, Bacteroidetes and Proteobacteria. (C) Butyric acid level. Results (C) were shown as the mean ± SD.


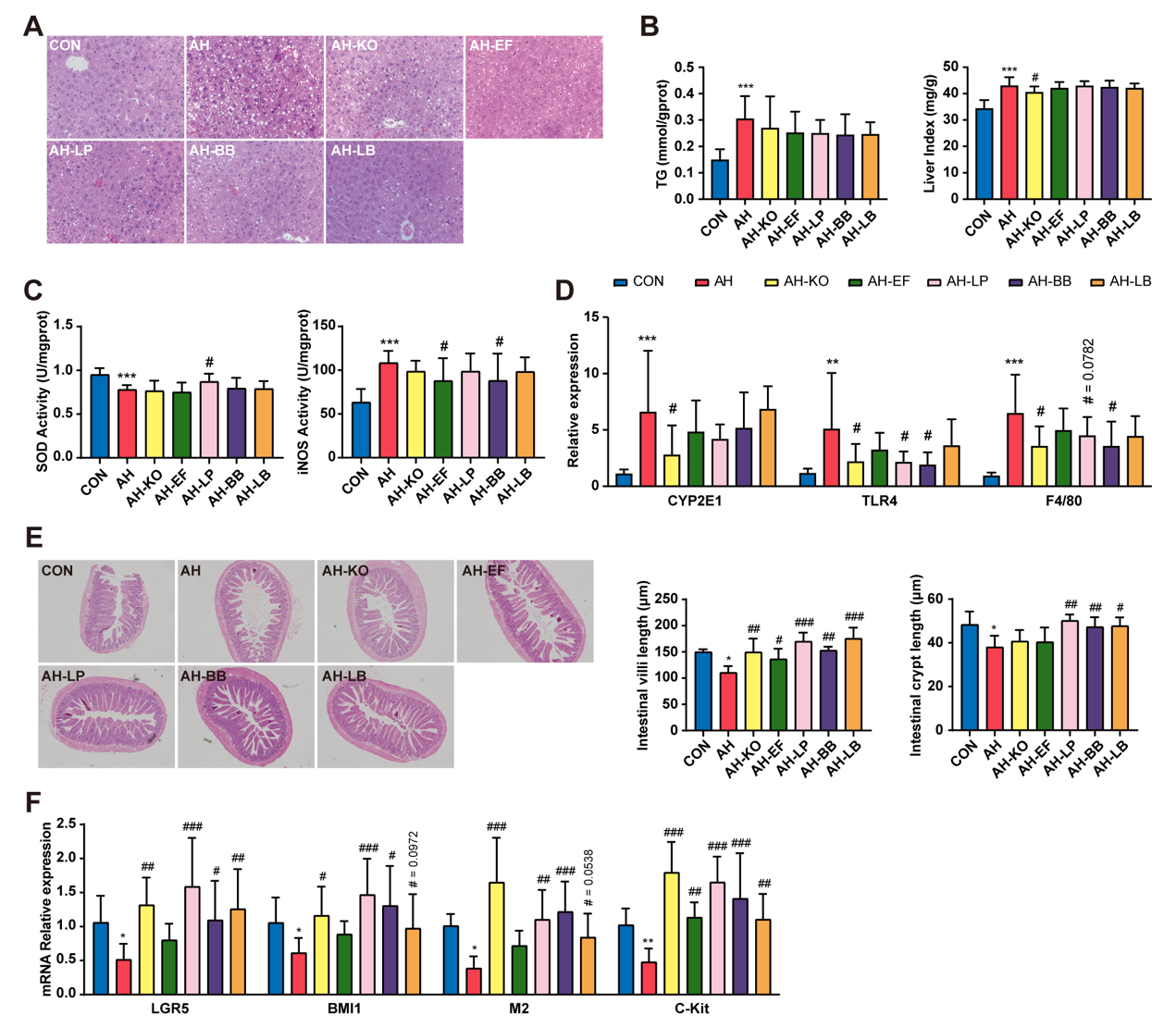


Fig. S11 Effect of microbiological samples administration on liver and small intestine

(A) Hepatic H&E staining (magnification: 400×). (B) Content of triglyceride in the liver and liver index. (C) SOD and iNOS activities in the liver. (D) Hepatic mRNA expression of CYP2E1, TLR4 and F4/80. (E) Small intestinal H&E staining (magnification: 100×), and the length of villi and crypts. (F) Small intestinal mRNA expression of LGR5, BMI1, M2 and C-kit. Results were shown as the mean ± SD. * *p* < 0.05, ** *p* < 0.01, *** *p* < 0.001 compared with CON group, and # *p* < 0.05, ## *p* < 0.01, ### *p* < 0.001 compared with AH group by ANOVA one-way statistical analysis.


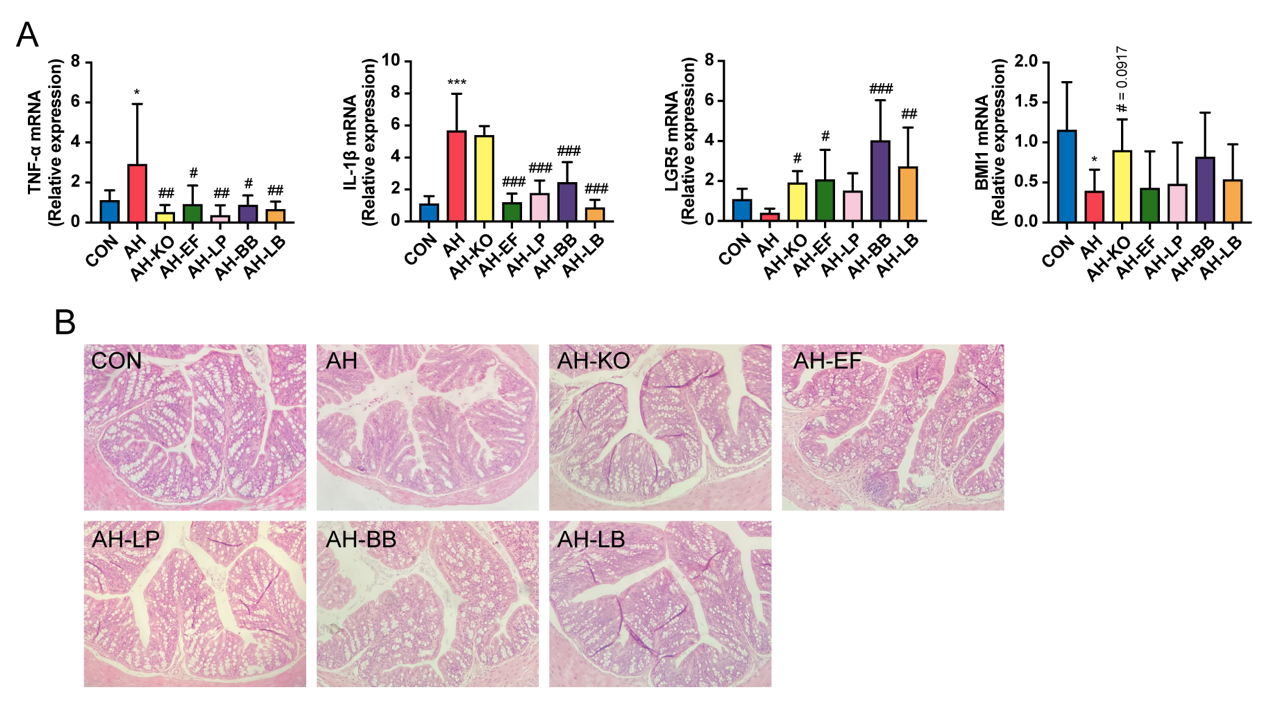


Fig. S12 Effect of microbiological samples administration on the cecum

(A) Cecal mRNA expression of TNF-α, IL-1β, LGR5 and BMI1. (B) Cecal H&E staining (magnification: 100×). Results were shown as the mean ± SD. * *p* < 0.05, *** *p* < 0.001 compared with CON group, and # *p* < 0.05, ## *p* < 0.01, ### *p* < 0.001 compared with AH group by ANOVA one-way statistical analysis.


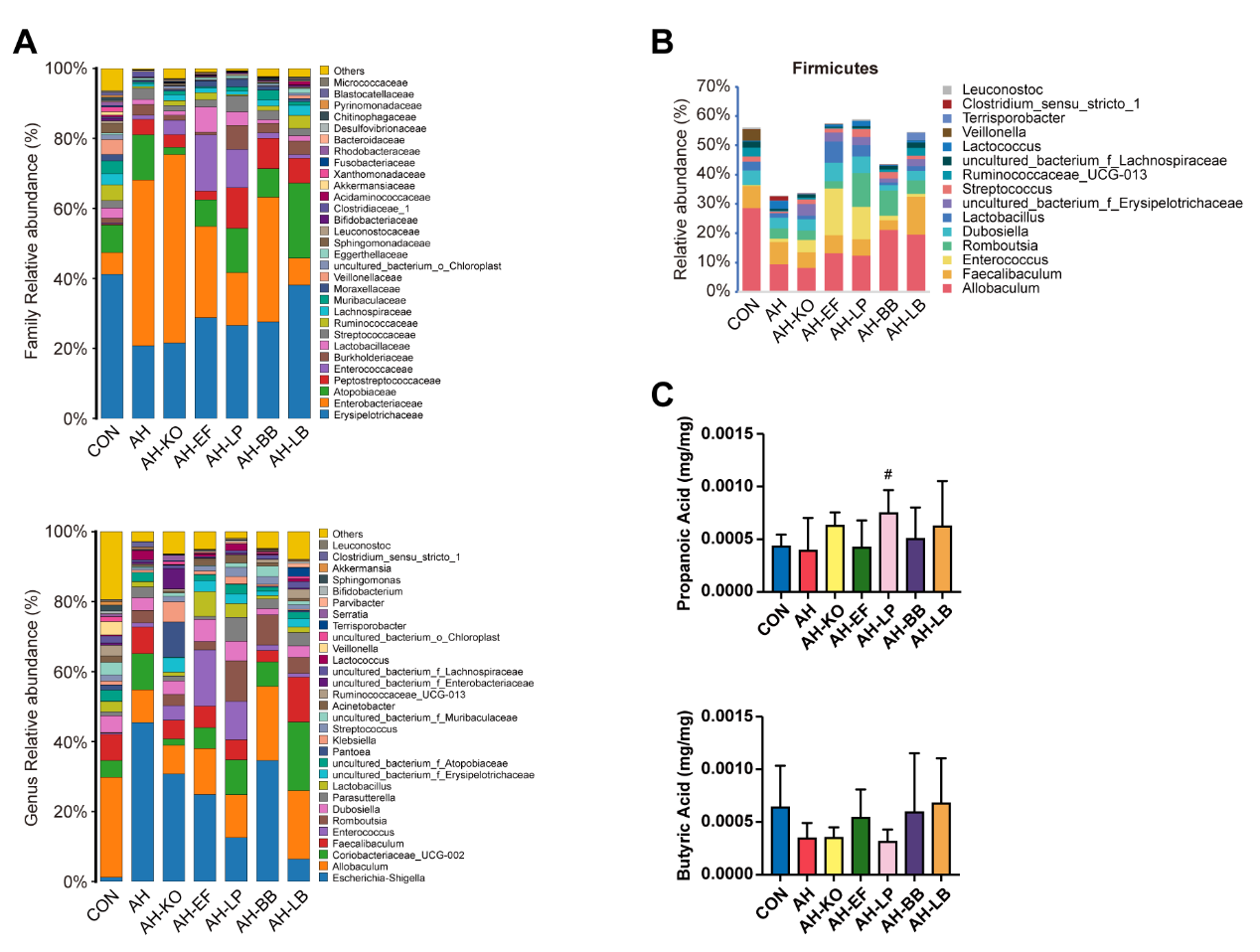


Fig. S13 Effect of microbiological samples administration on the bacterial composition and SCFA of the small intestine

(A) Relative abundance of microbiota at family and genus levels. (B) Bacterial composition at the genus level in Firmicutes. (C) SCFA levels. Results (C) were shown as the mean ± SD. # *p* < 0.05 compared with AH group by ANOVA one-way statistical analysis.


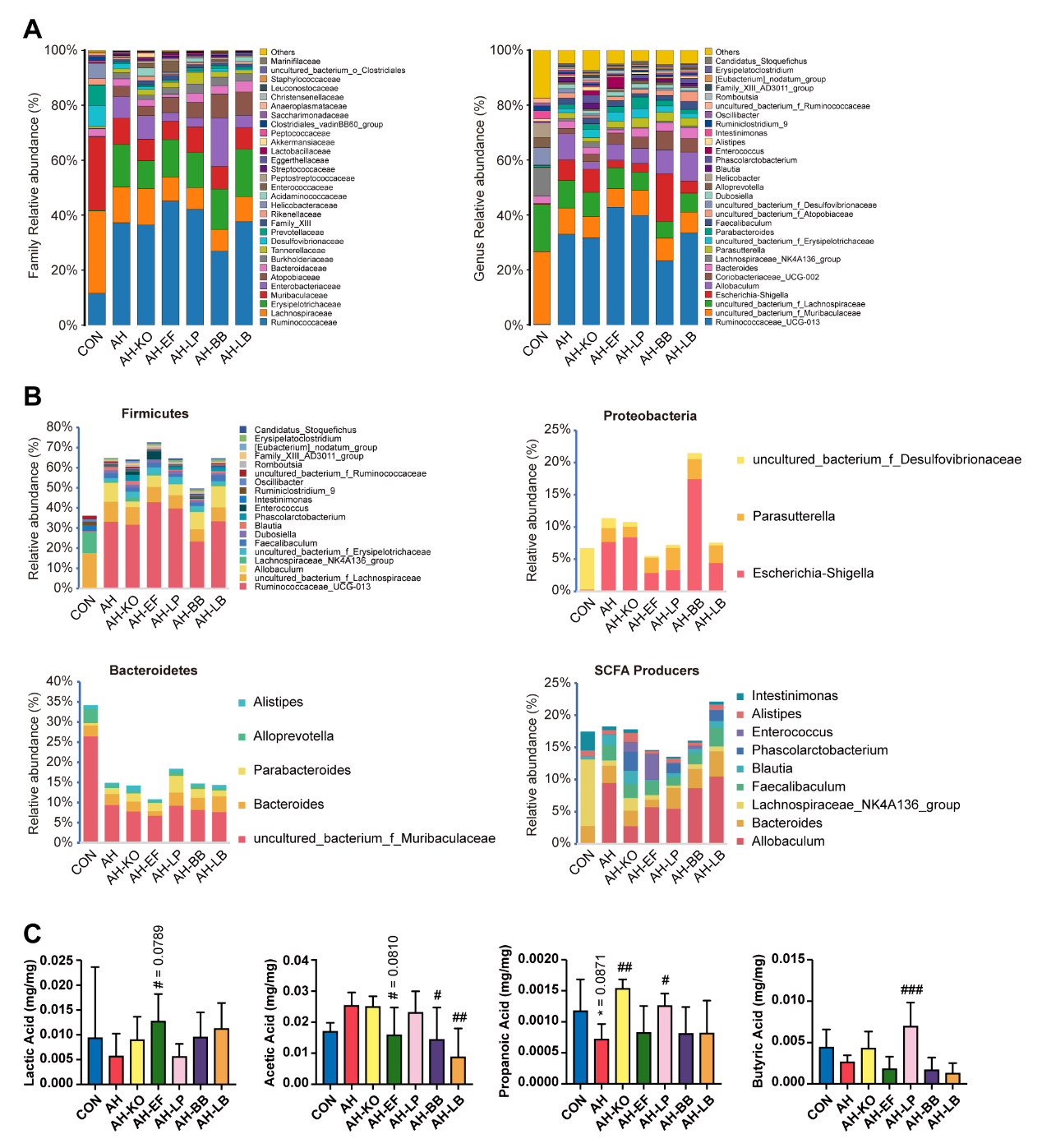


Fig. S14 Effect of microbiological samples administration on the bacterial composition and SCFA of the cecum

(A) Relative abundance of microbiota at family and genus levels. (B) Bacterial composition at the genus level in Firmicutes, Bacteroidetes and Proteobacteria, and relative abundance of SCFA producers. (C) SCFA levels. Results (C) were shown as the mean ± SD. # *p* < 0.05 and ## *p* < 0.01 compared with AH group by ANOVA one-way statistical analysis.


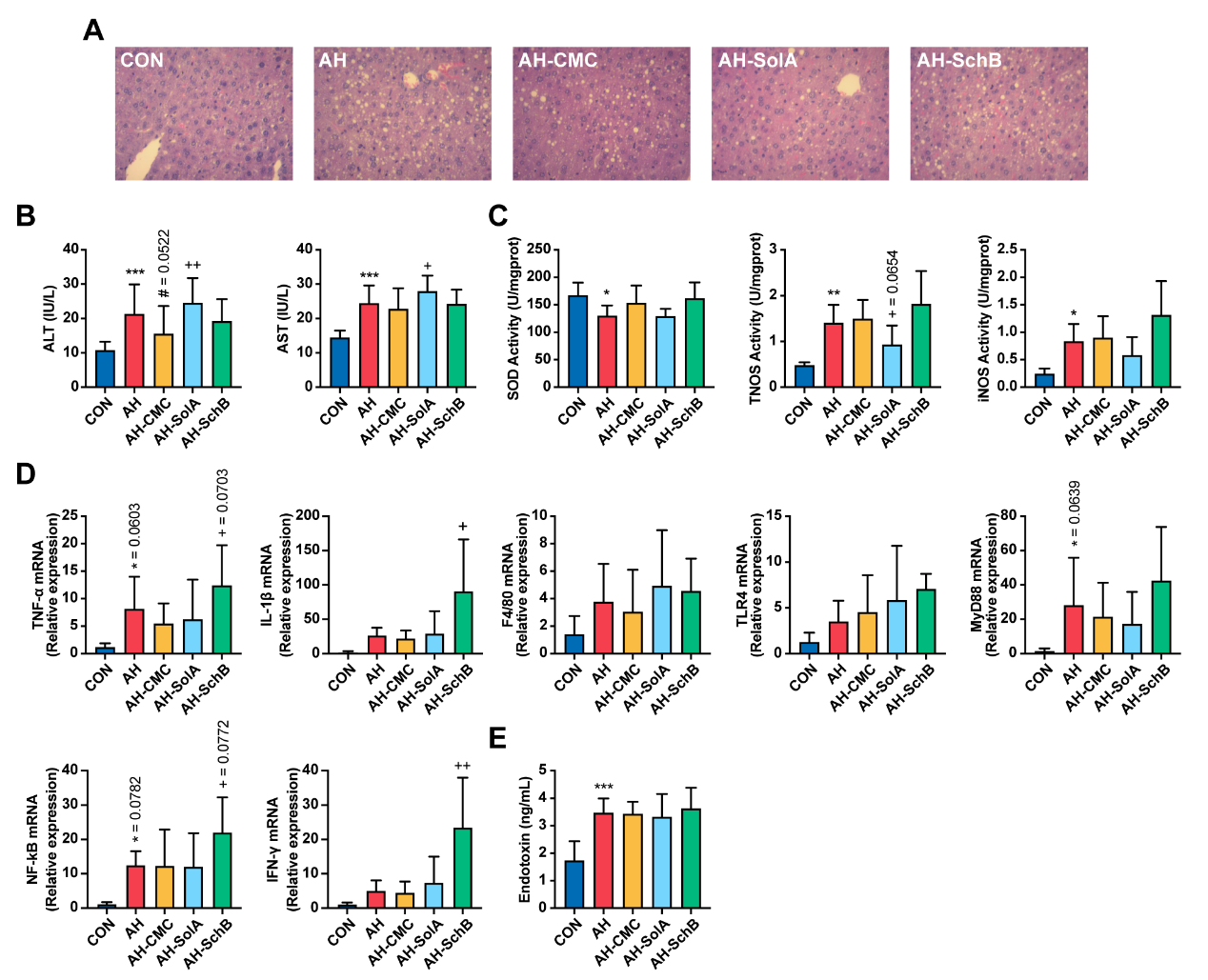


Fig. S15 Effects of schisandrol A and schisandrin B administration on AH

(A) Hepatic H&E staining (magnification: 400 ×). (B) ALT and AST in serum. (C) SOD, TNOS, and iNOS activities in the liver. (D) Hepatic mRNA expression of TNF-α, IL-1β, F4/80, TLR4, MyD88, NF-κB, and IFN-γ. (E) Serum LPS level. Results were shown as the mean ± SD. * *p*<0.05, ** *p*<0.01, *** *p*<0.001 compared with CON group, # *p*<0.05 compared with AH group, and + *p*<0.05, ++ *p*<0.01 compared with AHP group by ANOVA one-way statistical analysis.

Table S1. Content of effective components in *S. chinensis* extracts using chemical methods

|  | Content (%) | |
| --- | --- | --- |
|  | *S. chinensis* water extract | *S. chinensis* ethanol extract |
| Polysaccharide | 31.6642 ± 0.0102 | 21.0054 ± 0.0067 |
| Triterpenoid | 5.4502 ± 0.0004 | 11.2667 ± 0.0016 |
| Polyphenol | 0.2658 ± 0.0004 | 0.7177 ± 0.0014 |

Data were shown as the mean ± SD.

Table S2. Relative quantities of metabolites in the small intestinal contents

|  | CON | CSC | AH | AHSC |
| --- | --- | --- | --- | --- |
| Ethanol | 5.367 ± 1.047 | 5.384 ± 2.372 | 12.551 ± 0.173*** | 8.729 ± 1.402### |
| Glutamic acid | 12.898 ± 1.020 | 13.372 ± 0.283 | 12.345 ± 1.164 | 13.762 ± 0.622# |
| Glutamine | 0.706 ± 0.186 | 0.713 ± 0.052 | 0.844 ± 0.213 | 0.768 ± 0.085 |
| Aspartate | 1.152 ± 0.130 | 1.044 ± 0.091 | 1.168 ± 0.207 | 1.155 ± 0.119 |
| Asparagine | 2.558 ± 0.368 | 2.689 ± 0.197 | 2.461 ± 0.219 | 2.341 ± 0.098 |
| Glycine | 2.286 ± 0.369 | 2.191 ± 0.201 | 2.029 ± 0.108 | 1.930 ± 0.201 |
| Taurine | 3.805 ± 1.808 | 4.114 ± 0.739 | 2.476 ± 0.575 | 3.615 ± 0.979 |
| Tyrosine | 2.917 ± 0.291 | 3.164 ± 0.107 | 2.950 ± 0.865 | 2.841 ± 0.101 |
| Alanine | 8.967 ± 0.669 | 9.586 ± 0.466 | 8.524 ± 1.210 | 9.086 ± 0.386 |
| Valine | 7.162 ± 0.523 | 8.266 ± 0.208** | 7.689 ± 0.998 | 7.963 ± 0.224 |
| Leucine/Isoleucine | 4.474 ± 0.169 | 5.469 ± 0.345 | 4.212 ± 0.938 | 4.752 ± 0.321 |
| Threonine | 3.801 ± 0.259 | 4.163 ± 0.109* | 4.008 ± 0.289 | 3.876 ± 0.044 |
| Methionine | 3.049 ± 0.301 | 3.374 ± 0.061 | 3.473 ± 0.565 | 3.426 ± 0.141 |
| Phenylalanine | 3.556 ± 0.252 | 4.007 ± 0.132 | 3.675 ± 1.046 | 3.597 ± 0.126 |
| Tryptophan | 1.107 ± 0.247 | 1.031 ± 0.125 | 0.949 ± 0.133 | 1.063 ± 0.075 |
| Lysine | 0.295 ± 0.060 | 0.353 ± 0.037 | 0.369 ± 0.061 | 0.336 ± 0.028 |
| Total amino acid | 58.735 ± 4.270 | 63.537 ± 1.183 | 57.173 ± 7.585 | 60.512 ± 2.558 |

Data were shown as the mean ± SD (%). * *p*<0.05, ** *p*<0.01, *** *p*<0.001 compared with CON group, and # *p*<0.05, ### *p*<0.001 compared with AH group by ANOVA one-way statistical analysis.

Table S3. Relative quantities of metabolites in the cecal contents

|  | CON | CSC | AH | AHSC |
| --- | --- | --- | --- | --- |
| Ethanol | 11.210 ± 0.689 | 11.989 ± 1.684 | 19.434 ± 3.635*** | 21.401 ± 1.959*** |
| Glutamic acid | 7.843 ± 0.354 | 9.405 ± 1.000** | 7.901 ± 0.481 | 7.839 ± 0.457 |
| Glycine | 1.902 ± 0.640 | 2.288 ± 0.437 | 1.638 ± 0.506 | 1.169 ± 0.368 |
| Taurine | 2.640 ± 0.390 | 2.449 ± 0.051 | 2.078 ± 0.473* | 1.971 ± 0.203** |
| Tyrosine | 0.756 ± 0.077 | 0.748 ± 0.015 | 0.574 ± 0.138** | 0.584 ± 0.078** |
| Alanine | 2.028 ± 0.140 | 2.104 ± 0.022 | 1.788 ± 0.303 | 1.769 ± 0.077 |
| Leucine | 3.866 ± 0.300 | 4.094 ± 0.088 | 3.304 ± 0.657 | 3.541 ± 0.291 |
| Isoleucine | 1.904 ± 0.174 | 2.088 ± 0.140 | 1.689 ± 0.336 | 1.791 ± 0.124 |
| Methionine | 2.375 ± 0.156 | 2.758 ± 0.263** | 2.330 ± 0.148 | 2.462 ± 0.105 |
| Phenylalanine | 1.115 ± 0.095 | 1.128 ± 0.021 | 0.896 ± 0.208* | 0.907 ± 0.119* |
| Lysine | 3.673 ± 0.405 | 3.797 ± 0.026 | 3.064 ± 0.620* | 2.977 ± 0.061* |
| Total amino acid | 28.101 ± 1.718 | 30.860 ± 1.796 | 25.262 ± 1.918 | 25.009 ± 0.482 |

Data were shown as the mean ± SD (%). * *p*<0.05, ** *p*<0.01, *** *p*<0.001 compared with CON group by ANOVA one-way statistical analysis.

Table S4. Antibacterial experiment of *S. chinensis* extracts

| Bacterial strains | Inhibitive diameter (mm) | | | |
| --- | --- | --- | --- | --- |
|  | Control | SCE (mix) | SCWE | SCEE |
| *Salmonella enterica subsp.*  *enterica serovar typhimurium* | 6.00 | 13.11±2.250 | 13.9±0.1.136 | 6.00 |
| *Enterobacter cloacae* | 6.00 | 13.22±1.155 | 10.72±0.4843 | 6.00 |
| *Vibrio cholerae* | 6.00 | 14.42±1.431 | 24.56±1.020 | 13.43±0.0.7924 |
| *Staphylococcus aureus* | 6.00 | 12.41±1.494 | 13.69±1.448 | 6.00 |
| *Vibrio parahaemolyticus* | 6.00 | 22.52±2.270 | 26.71±1.488 | 11.74±1.005 |
| *Pseudomonas aeruginosa* P7 | 6.00 | 6.00 | 8.547±0.3881 | 6.00 |
| *Pseudomonas aeruginosa* P11 | 6.00 | 14.47±0.6052 | 17.56±0.4507 | 8.027±0.2181 |
| *Pseudomonas aeruginosa* P16 | 6.00 | 15.17±1.092 | 17.98±2.232 | 7.66±0.4445 |
| *Shigella flexneri* | 6.00 | 13.21±1.828 | 15.88±2.226 | 6.00 |
| *Klebsiella oxytoca* | 6.00 | 12.89±0.6816 | 16.11±0.8118 | 6.00 |

Data were shown as the mean ± SD.

Table S5. Bacterial growth test of *S. chinensis* extracts

| Strains (10^8^ CFU/mL) | CON | E0.5 | E1 | E2 | W0.5 | W1 | W2 | M0.5 | M1 | M2 |
| --- | --- | --- | --- | --- | --- | --- | --- | --- | --- | --- |
| *Enterococcus faecalis* (13 h) | 17.977±0.171 | 18.979±0.562 | 17.977±1.246 | 18.271±0.488 | 17.977±1.001 | 14.109±1.783 | 6.597±0.147 | 17.027±0.488 | 15.283±1.050 | 7.702±0.293 |
| *Enterococcus faecium* (12 h) | 2.792±0.037 | 2.835±0.172 | 2.705±0.053 | 2.763±0.078 | 2.572±0.176 | 2.546±0.131 | 1.212±0.107 | 2.763±0.103 | 2.354±0.066 | 1.319±0.340 |
| *Lactobacillus rhamnosu* (12 h) | 8.231±0.161 | 7.921±0.133 | 8.129±0.283 | 7.961±0.000 | 8.188±0.133 | 8.262±0.216 | 7.870±0.028 | 8.121±0.305 | 8.019±0.050 | 7.878±0.105 |
| *Lactobacillus salivarius* (12 h) | 4.263±0.113 | 4.298±0.045 | 4.275±0.071 | 4.275±0.059 | 4.452±0.059 | 4.307±0.056 | 4.208±0.107 | 4.300±0.024 | 4.204±0.048 | 4.385±0.131 |
| *Lactobacillus paracasei* (12 h) | 4.805±0.058 | 4.787±0.008 | 4.481±0.482 | 4.772±0.037 | 4.522±0.308 | 4.611±0.033 | 4.175±0.108 | 4.611±0.083 | 4.449±0.021 | 3.902±0.087 |
| *Lactobacillus casei* (13 h) | 3.544±0.423 | 3.473±0.000 | 3.622±0.211 | 3.295±0.040 | 3.434±0.287 | 3.103±0.372 | 2.427±0.060 | 3.437±0.423 | 3.420±0.247 | 2.377±0.010 |
| *Lactobacillus reuteri* (13 h) | 0.333±0.007 | 0.313±0.019 | 0.340±0.005 | 0.310±0.006 | 0.404±0.021 | 0.545±0.038 | 0.480±0.023 | 0.460±0.035 | 0.606±0.028 | 0.660±0.042 |
| *Lactobacillus plantarum* (12 h) | 4.978±0.114 | 5.042±0.197 | 4.983±0.265 | 5.106±0.091 | 5.278±0.038 | 5.149±0.061 | 4.911±0.178 | 5.165±0.053 | 5.018±0.034 | 4.900±0.140 |
| *Lactobacillus bulgaricus* (13 h) | 2.836±0.111 | 2.900±0.020 | 2.876±0.054 | 2.891±0.000 | 2.874±0.008 | 2.743±0.036 | 2.587±0.046 | 2.907±0.006 | 2.700±0.012 | 2.647±0.050 |
| *Streptococcus thermophiles* (13 h) | 0.226±0.020 | 0.235±0.008 | 0.208±0.006 | 0.187±0.005 | 0.120±0.002 | 0.058±0.003 | 0.059±0.002 | 0.119±0.007 | 0.064±0.023 | 0.056±0.004 |
| *Bifidobacterium adolescentis* (12 h) | 0.376±0.010 | 0.386±0.010 | 0.368±0.025 | 0.386±0.003 | 0.449±0.040 | 0.443±0.022 | 0.255±0.013 | 0.511±0.037 | 0.497±0.050 | 0.289±0.003 |
| *Bifidobacterium breve* (12 h) | 0.294±0.021 | 0.283±0.018 | 0.308±0.025 | 0.266±0.028 | 0.266±0.002 | 0.288±0.010 | 0.236±0.002 | 0.274±0.013 | 0.238±0.012 | 0.213±0.021 |
| *Bifidobacterium longum* (12 h) | 0.571±0.011 | 0.535±0.011 | 0.500±0.031 | 0.510±0.006 | 0.480±0.066 | 0.480±0.003 | 0.371±0.020 | 0.527±0.017 | 0.506±0.063 | 0.464±0.014 |
| *Bifidobacterium bifidum* (12 h) | 0.327±0.027 | 0.317±0.009 | 0.370±0.034 | 0.304±0.009 | 0.342±0.005 | 0.322±0.005 | 0.213±0.018 | 0.354±0.004 | 0.373±0.027 | 0.207±0.002 |

Data were shown as the mean ± SD.

Table S6. Primer sequences for real-time reverse transcription polymerase chain reaction

| Primer | Position | Forward sequence (5'-3') | Reverse sequence (5'-3') |
| --- | --- | --- | --- |
| m-*18S* | Liver | ACGGAAGGGCACCACCAGGA | CACCACCACCCACGGAATCG |
| m-*F4/80* | Liver | TGACTCACCTTGTGGTCCTAA | CTTCCCAGAATCCAGTCTTTCC |
| m-*Mcp-1* | Liver | TTAAAAACCTGGATCGGAACCAA | GCATTAGCTTCAGATTTACGGGT |
| m-*Tlr4* | Liver | ATGGCATGGCTTACACCACC | GAGGCCAATTTTGTCTCCACA |
| m-*Myd88* | Liver | GTATCCTGCGGTTCATCAC | TCAGTCTCATCTTCCCCTCT |
| m-*Tnf-α* | Liver | AGACCCTCACACTCAGATCA | TCTTTGAGATCCATGCCGTTG |
| m-*Il-1β* | Liver | TCCATGAGCTTTGTACAAGGA | AGCCCATACTTTAGGAAGACA |
| m-*Ifn-γ* | Liver | TCAAGTGGCATAGATGTGGAAGAA | TGGCTCTGCAGGATTTTCATG |
| m-*Adh1* | Liver | GCAAAGCTGCGGTGCTATG | TCACACAAGTCACCCCTTCTC |
| m-*Aldh1a1* | Liver | AGGCCCTCAGATTGACAAGGA | GTTGCACTGGTCCAAATATCT |
| m-*Aldh1b1* | Liver | AGCGCGATTCGGAGCCTCA | TGACCGCATCATGCCACTCGT |
| m-*Aldh2* | Liver | AGGTCTTCTGCAACCAGATCT | AGATGCATCCATGCGGCG |
| m-*Cat* | Liver | GCGGATTCCTGAGAGAGTGG | TGTGGAGAATCGAACGGCAA |
| m-*Cyp2e1* | Liver | CGTTGCCTTGCTTGTCTGGA | AAGAAAGGAATTGGGAAAGGTCC |
| m-*28S* | Ileum | TTGAAAATCCGGGGGAGAG | ACATTGTTCCAACATGCCAG |
| m-*Lgr5* | Ileum | CCTGCTTGACTTTGAGGAAGAC | ATGTTCACTGCTGCGATGAC |
| m-*Bmi1* | Ileum | AGCAGAAATGCATCGAACAA | CCTAACCAGATGAAGTTGCTG |
| m-*C-kit* | Ileum | GCCAAGGGCATGGCGTTCCTCGCCTCCAAG | TGTCCGAGATCTGCTTCTCAATAAGTTGGAC |
| m-*M2* | Ileum | TAAAGTCAACCGCCACCTTC | ATAACGGAGGCATTGCTGAC |

**References**

[1] Xu G, Niu J, Yuan G, Bai Y, Li H, Sheng Y, et al. Determination of eight lignans in Schisandra chinensis and Schisandra sphenanthera. Bangl J Pharmacol. 2016;11(Special).
