# Supplementary material for: Intestinal Microbiota Contributes to the Improvement of Alcoholic Hepatitis in Mice Treated With Schisandra chinensis Extract
Source: Front Nutr. 2022 Feb 18;9:822429. doi: 10.3389/fnut.2022.822429 (PMC8894320; doi:10.3389/fnut.2022.822429)
Supplement: Supplementary file 3 [file Table_2.DOCX]

Some of the main components of *Schisandra chinensis* extracts based on LC-MS and their bioactivities.

| Name | Class | Bioactivity | Host/Cell | Reference |
| --- | --- | --- | --- | --- |
| Benzoic acid | Phenols | Improving intestinal barrier functions | Piglets | [1,2] |
| Gossypol | Phenols | Anticancer activity | Human colon cancer cells (COLO 225),  human triple-negative breast cancer cells,  human prostate cancer cells | [3-5] |
|  |  | Anti-inflammatory activity | Mice | [6] |
|  |  | Antifungal activity | - | [7] |
| Gallic acid | Phenols | Antimicrobial activity | - | [8] |
|  |  | Anticancer activity | Human non‑small cell lung cancer cells | [9] |
|  |  | Cardioprotection | Rats | [10] |
|  |  | Anti-inflammatory activity | Human hepatoma cell line HepG2,  murine hepatoma cell line Hepa 1-6,  murine macrophage cell line RAW 264 | [11] |
| Dihydromyricetin | Flavonoids | Improving liver diseases | (Multi) | [12] |
|  |  | Protecting intestine | Mice | [13] |
| Myricetin | Flavonoids | Anticancer activity | (Multi) | [14] |
|  |  | Antimicrobial, antioxidant, anti-inflammatory and immunomodulatory activities | (Multi) | [15] |
| Chalcone | Flavonoids | Anticancer activity | (Multi) | [16] |
|  |  | Hepatoprotive activity | (Multi) | [17] |
| Name | Class | Bioactivity | Host/Cell | Reference |
| Quercetin | Flavonoids | Attenuating muscle damage and pain, accelerating recovery | Clinical Trial | [18] |
|  |  | Improving metabolic diseases | (Multi) | [19] |
|  |  | Anti-obesity | Mice | [20] |
|  |  | Antimicrobial, antioxidant, anti-inflammatory and liver-protective activities | (Multi) | [21] |
|  |  | Neuroprotective effects | (Multi) | [22] |
| Rutin | Flavonoids | Hepatoprotive activity | Mice | [23] |
|  |  | Protecting intestine | Mice | [24] |
|  |  | Antioxidant and anti-inflammatory activities | Mice | [25] |
| Kaempferol | Flavonoids | Protecting intestine | Mice | [26] |
| Asiatic acid | Terpenoids | Anti-obesity | Rats | [27] |
|  |  | Anti-inflammatory activity | Mice | [28] |
| alpha-Hederin | Terpenoids | Anticancer activity | Human gastric cancer cell lines (HGC-27 and SGC-7901) | [29] |
| Betulinic acid | Terpenoids | Anticancer activity | (Multi) | [30] |
| Schisandrol B | Phenylpropanoids | Hepatoprotive activity | Mice | [31,32] |
| Schizandrin A | Phenylpropanoids | Anticancer activity | Human breast cancer cell line MDA-MB-231,  Human colorectal cancer cell lines DLD1, RKO, SW480, SW620 | [33,34] |
|  |  | Anti-inflammatory activity | Mice | [35] |
|  |  | Hepatoprotive activity | Mice | [36] |
| Cryptochlorogenic acid | Phenylpropanoids | Antioxidant and anti-inflammatory activities | RAW 264.7 murine macrophage cells | [37] |
| Name | Class | Bioactivity | Host/Cell | Reference |
| Chlorogenic acid | Phenylpropanoids | Improving diabetes mellitus | (Multi) | [38] |
|  |  | Anti-inflammatory activity | (Multi) | [39] |
|  |  | Hepatoprotive activity | Mice | [40] |
|  |  | Protecting intestine | Rats | [41] |
| Dehydrodiisoeugenol | Lignans | Anticancer activity | Human colorectal cancer cell lines (HCT 116 and SW620) | [42] |
| Schisandrin C | Lignans | Antioxidant activity | Mice | [43] |
|  |  | Antioxidant and anti-inflammatory activities | Human dental pulp cells | [44] |
| Schisantherin A | Lignans | Ameliorating liver fibrosis | Mice | [45] |
|  |  | Anticancer activity | Human gastric cancer cell lines (MKN45 and SGC7901) | [46] |
|  |  | Anti-inflammatory activity | Human chondrocytes | [47] |
| Schizandrol B | Lignans | Hepatoprotive activity | Mice | [48,49] |
| L-Isoleucine | Amino acid derivatives | Immunoregulation | Piglets | [50] |
| Arginine | Amino acid derivatives | Improving cardiovascular disorders | (Multi) | [51] |
|  |  | Decreasing oxidative stress | Clinical Trial | [52] |
| Nicotinamide | Alkaloids | Ameliorating acne | Clinical Trial | [53] |
|  |  | Preventing cancers | (Multi) | [54,55] |
| Stachydrine | Alkaloids | Improving cardiovascular disorders, preventing cancers, neuroprotective and anti-inflammatory effects | (Multi) | [56] |
| Citrate | Organic acids and derivatives | Anticancer activity | Mice | [57,58] |
|  |  | Treating calcium containing kidney stones | Clinical Trial | [59] |
| Name | Class | Bioactivity | Host/Cell | Reference |
| Vasicine | Alkaloids | Antioxidant and anti-inflammatory activities | Rats | [60] |
| Shikimic acid | Organic acids and derivatives | Anti-inflammatory activity | Murine macrophage cell line RAW 264.7 | [61] |
| Pantothenic acid | Carboxylic acids and derivatives | Hepatoprotive activity | Rats | [62] |

**References**

[1] Halas D, Hansen CF, Hampson DJ, Mullan BP, Wilson RH, Pluske JR. Effect of dietary supplementation with inulin and/or benzoic acid on the incidence and severity of post-weaning diarrhoea in weaner pigs after experimental challenge with enterotoxigenic Escherichia coli. Arch Anim Nutr. 2009;63(4):267-280. doi: 10.1080/17450390903020414.

[2] Chen JL, Zheng P, Zhang C, Yu B, He J, Yu J, et al. Benzoic acid beneficially affects growth performance of weaned pigs which was associated with changes in gut bacterial populations, morphology indices and growth factor gene expression. J Anim Physiol Anim Nutr (Berl). 2017;101:1137-1146. doi: 10.1111/jpn.12627.

[3] Cao H, Sethumadhavan K, Cao F, Wang T. Gossypol decreased cell viability and down-regulated the expression of a number of genes in human colon cancer cells. Sci Rep. 2021;11:5922. doi: 10.1038/s41598-021-84970-8.

[4] Messeha SS, Zarmouh NO, Mendonca P, Alwagdani H, Cotton C, Soliman KFA. Effects of gossypol on apoptosisrelated gene expression in racially distinct triplenegative breast cancer cells. Oncol Rep. 2019;42(2):467-478. doi: 10.3892/or.2019.7179.

[5] Pang X, Wu Y, Wu Y, Lu B, Chen J, Wang J, et al. (-)-Gossypol suppresses the growth of human prostate cancer xenografts via modulating VEGF signaling-mediated angiogenesis. Mol Cancer Ther. 2011;10(5):795-805. doi: 10.1158/1535-7163.MCT-10-0936.

[6] Huo M, Gao R, Jiang L, Cui X, Duan L, Deng X, et al. Suppression of LPS-induced inflammatory responses by gossypol in RAW 264.7 cells and mouse models. Int Immunopharmacol. 2013;15(2):442-449. doi: 10.1016/j.intimp.2013.01.008.

[7] Mellon JE, Zelaya CA, Dowd MK, Beltz SB, Klich MA. Inhibitory effects of gossypol, gossypolone, and apogossypolone on a collection of economically important filamentous fungi. J Agric Food Chem. 2012;60:2740-2745. doi: 10.1021/jf2044394.

[8] Wang Q, Leong WF, Elias RJ, Tikekar RV. UV-C irradiated gallic acid exhibits enhanced antimicrobial activity via generation of reactive oxidative species and quinone. Food Chem. 2019;287:303-312. doi: 10.1016/j.foodchem.2019.02.041.

[9] Zhang T, Ma L, Wu P, Li W, Li T, Gu R, et al. Gallic acid has anticancer activity and enhances the anticancer effects of cisplatin in nonsmall cell lung cancer A549 cells via the JAK/STAT3 signaling pathway. Oncol Rep. 2019;41(3):1779-1788. doi: 10.3892/or.2019.6976.

[10] Priscilla DH, Prince PS. Cardioprotective effect of gallic acid on cardiac troponin-T, cardiac marker enzymes, lipid peroxidation products and antioxidants in experimentally induced myocardial infarction in Wistar rats. Chem Biol Interact. 2009;179:118-124. doi: 10.1016/j.cbi.2008.12.012.

[11] Tanaka M, Sato A, Kishimoto Y, Mabashi-Asazuma H, Kondo K, Iida K. Gallic Acid Inhibits Lipid Accumulation via AMPK Pathway and Suppresses Apoptosis and Macrophage-Mediated Inflammation in Hepatocytes. Nutrients. 2020;12(5):1479. doi: 10.3390/nu12051479.

[12] Chen J, Wang X, Xia T, Bi Y, Liu B, Fu J, et al. Molecular mechanisms and therapeutic implications of dihydromyricetin in liver disease. Biomed Pharmacother. 2021;142:111927. doi: 10.1016/j.biopha.2021.111927.

[13] Dong S, Zhu M, Wang K, Zhao X, Hu L, Jing W, et al. Dihydromyricetin improves DSS-induced colitis in mice via modulation of fecal-bacteria-related bile acid metabolism. Pharmacol Res. 2021;171:105767. doi: 10.1016/j.phrs.2021.105767.

[14] Jiang M, Zhu M, Wang L, Yu S. Anti-tumor effects and associated molecular mechanisms of myricetin. Biomed Pharmacother. 2019;120:109506. doi: 10.1016/j.biopha.2019.109506.

[15] Song X, Tan L, Wang M, Ren C, Guo C, Yang B, et al. Myricetin: A review of the most recent research. Biomed Pharmacother. 2021;134:111017. doi: 10.1016/j.biopha.2020.111017.

[16] Gao F, Huang G, Xiao J. Chalcone hybrids as potential anticancer agents: Current development, mechanism of action, and structure-activity relationship. Med Res Rev. 2020;40(5):2049-2084. doi: 10.1002/med.21698.

[17] Karimi-Sales E, Mohaddes G, Alipour MR. Chalcones as putative hepatoprotective agents: Preclinical evidence and molecular mechanisms. Pharmacol Res. 2018;129:177-187. doi: 10.1016/j.phrs.2017.11.022.

[18] Bazzucchi I, Patrizio F, Ceci R, Duranti G, Sabatini S, Sgrò P, et al. Quercetin Supplementation Improves Neuromuscular Function Recovery from Muscle Damage. Nutrients. 2020;12(9):2850. doi: 10.3390/nu12092850.

[19] Yi H, Peng H, Wu X, Xu X, Kuang T, Zhang J, et al. The Therapeutic Effects and Mechanisms of Quercetin on Metabolic Diseases: Pharmacological Data and Clinical Evidence. Oxid Med Cell Longev. 2021;2021:6678662. doi: 10.1155/2021/6678662.

[20] Zhao L, Zhu X, Xia M, Li J, Guo AY, Zhu Y, et al. Quercetin Ameliorates Gut Microbiota Dysbiosis That Drives Hypothalamic Damage and Hepatic Lipogenesis in Monosodium Glutamate-Induced Abdominal Obesity. Front Nutr. 2021;8:671353. doi:10.3389/fnut.2021.671353.

[21] Batiha GE, Beshbishy AM, Ikram M, Mulla ZS, El-Hack MEA, Taha AE, et al. The Pharmacological Activity, Biochemical Properties, and Pharmacokinetics of the Major Natural Polyphenolic Flavonoid: Quercetin. Foods. 2020;9(3):374. doi: 10.3390/foods9030374.

[22] Alvarez-Arellano L, Salazar-Garcia M, Corona JC. Neuroprotective Effects of Quercetin in Pediatric Neurological Diseases. Molecules. 2020;25(23):5597. doi: 10.3390/molecules25235597.

[23] Li S, Li J, Pan R, Cheng J, Cui Q, Chen J, et al. Sodium rutin extends lifespan and health span in mice including positive impacts on liver health. Br J Pharmacol. 2021;10. doi: 10.1111/bph.15410.

[24] Fideles LS, de Miranda JAL, Martins CDS, Barbosa MLL, Pimenta HB, Pimentel PVS, et al. Role of Rutin in 5-Fluorouracil-Induced Intestinal Mucositis: Prevention of Histological Damage and Reduction of Inflammation and Oxidative Stress. Molecules. 2020;25(12):2786. doi: 10.3390/molecules25122786.

[25] Ma JQ, Liu CM, Yang W. Protective effect of rutin against carbon tetrachloride-induced oxidative stress, inflammation and apoptosis in mouse kidney associated with the ceramide, MAPKs, p53 and calpain activities. Chem Biol Interact. 2018;286:26-33. doi: 10.1016/j.cbi.2018.03.003.

[26] Qu Y, Li X, Xu F, Zhao S, Wu X, Wang Y, et al. Kaempferol Alleviates Murine Experimental Colitis by Restoring Gut Microbiota and Inhibiting the LPS-TLR4-NF-κB Axis. Front Immunol. 2021;12:679897. doi: 10.3389/fimmu.2021.679897.

[27] Uddandrao VVS, Rameshreddy P, Brahmanaidu P, Ponnusamy P, Balakrishnan S, Ramavat RN, et al. Antiobesity efficacy of asiatic acid: down-regulation of adipogenic and inflammatory processes in high fat diet induced obese rats. Arch Physiol Biochem. 2020;126(5):453-462. doi: 10.1080/13813455.2018.1555668.

[28] Yang C, Guo Y, Huang TS, Zhao J, Huang XJ, Tang HX, et al. Asiatic acid protects against cisplatin-induced acute kidney injury via anti-apoptosis and anti-inflammation. Biomed Pharmacother. 2018;107:1354-1362. doi: 10.1016/j.biopha.2018.08.126.

[29] Wang J, Deng H, Zhang J, Wu D, Li J, Ma J, et al. alpha-Hederin induces the apoptosis of gastric cancer cells accompanied by glutathione decrement and reactive oxygen species generation via activating mitochondrial dependent pathway. Phytother Res. 2020;34(3):601-611. doi: 10.1002/ptr.6548.

[30] Zhang DM, Xu HG, Wang L, Li YJ, Sun PH, Wu XM, et al. Betulinic Acid and its Derivatives as Potential Antitumor Agents. Med Res Rev. 2015;35(6):1127-1155. doi: 10.1002/med.21353.

[31] Zeng H, Jiang Y, Chen P, Fan X, Li D, Liu A, et al. Schisandrol B protects against cholestatic liver injury through pregnane X receptors. Br J Pharmacol. 2017;174:672-688. doi: 10.1111/bph.13729.

[32] Jiang YM, Wang Y, Tan HS, Yu T, Fan XM, Chen P, et al. Schisandrol B protects against acetaminophen-induced acute hepatotoxicity in mice via activation of the NRF2/ARE signaling pathway. Acta Pharmacol Sin. 2016;37(3):382-389. doi: 10.1038/aps.2015.120.

[33] Yan H, Guo M. Schizandrin A inhibits cellular phenotypes of breast cancer cells by repressing miR-155. IUBMB Life. 2020;72(8):1640-1648. doi: 10.1002/iub.2329.

[34] Chen BC, Tu SL, Zheng BA, Dong QJ, Wan ZA, Dai QQ. Schizandrin A exhibits potent anticancer activity in colorectal cancer cells by inhibiting heat shock factor 1. Biosci Rep. 2020;40. doi: 10.1042/BSR20200203.

[35] Cui L, Zhu W, Yang Z, Song X, Xu C, Cui Z, et al. Evidence of anti-inflammatory activity of Schizandrin A in animal models of acute inflammation. Naunyn Schmiedebergs Arch Pharmacol. 2020;393(11):2221-2229. doi: 10.1007/s00210-020-01837-x.

[36] Jeong MJ, Kim SR, Jung UJ. Schizandrin A supplementation improves nonalcoholic fatty liver disease in mice fed a high-fat and high-cholesterol diet. Nutr Res. 2019;64:64-71. doi: 10.1016/j.nutres.2019.01.001.

[37] Zhao XL, Yu L, Zhang SD, Ping K, Ni HY, Qin XY, et al. Cryptochlorogenic acid attenuates LPS-induced inflammatory response and oxidative stress via upregulation of the Nrf2/HO-1 signaling pathway in RAW 264.7 macrophages. Int Immunopharmacol. 2020;83:106436. doi: 10.1016/j.intimp.2020.106436.

[38] Yan Y, Zhou X, Guo K, Zhou F, Yang H. Use of Chlorogenic Acid against Diabetes Mellitus and Its Complications. J Immunol Res. 2020;2020:9680508. doi: 10.1155/2020/9680508.

[39] Bagdas D, Gul Z, Meade JA, Cam B, Cinkilic N, Gurun MS. Pharmacologic Overview of Chlorogenic Acid and its Metabolites in Chronic Pain and Inflammation. Curr Neuropharmacol. 2020;18(3):216-228. doi: 10.2174/1570159X17666191021111809.

[40] Shi A, Li T, Zheng Y, Song Y, Wang H, Wang N, Dong L, et al. Chlorogenic Acid Improves NAFLD by Regulating gut Microbiota and GLP-1. Front Pharmacol. 2021;12:693048. doi: 10.3389/fphar.2021.693048.

[41] Xie MG, Fei YQ, Wang Y, Wang WY, Wang Z. Chlorogenic Acid Alleviates Colon Mucosal Damage Induced by a High-Fat Diet via Gut Microflora Adjustment to Increase Short-Chain Fatty Acid Accumulation in Rats. Oxid Med Cell Longev. 2021;2021:3456542. doi: 10.1155/2021/3456542.

[42] Li C, Zhang K, Pan G, Ji H, Li C, Wang X, et al. Dehydrodiisoeugenol inhibits colorectal cancer growth by endoplasmic reticulum stress-induced autophagic pathways. J Exp Clin Cancer Res. 2021;40:125. doi: 10.1186/s13046-021-01915-9.

[43] Han J, Shi X, Du Y, Shi F, Zhang B, Zheng Z, et al. Schisandrin C targets Keap1 and attenuates oxidative stress by activating Nrf2 pathway in Ang II-challenged vascular endothelium. Phytother Res. 2019;33(3):779-790. doi: 10.1002/ptr.6271.

[44] Takanche JS, Lee YH, Kim JS, Kim JE, Han SH, Lee SW, et al. Anti-inflammatory and antioxidant properties of Schisandrin C promote mitochondrial biogenesis in human dental pulp cells. Int Endod J. 2018;51(4):438-447. doi: 10.1111/iej.12861.

[45] Wang H, Che J, Cui K, et al. Schisantherin A ameliorates liver fibrosis through TGF-beta1mediated activation of TAK1/MAPK and NF-kappaB pathways in vitro and in vivo. Phytomedicine. 2021;88:153609. https://doi.org/10.1016/j.phymed.2021.153609.

[46] Wang H, Che J, Cui K, Zhuang W, Li H, Sun J, et al. Schisantherin A induces cell apoptosis through ROS/JNK signaling pathway in human gastric cancer cells. Biochem Pharmacol. 2020;173:113673. doi: 10.1016/j.phymed.2021.153609.

[47] Liao S, Zhou K, Li D, Xie X, Jun F, Wang J. Schisantherin A suppresses interleukin-1beta-induced inflammation in human chondrocytes via inhibition of NF-kappaB and MAPKs activation. Eur J Pharmacol. 2016;780:65-70. doi: 10.1016/j.ejphar.2016.03.032.

[48] Zeng H, Jiang Y, Chen P, Fan X, Li D, Liu A, et al. Schisandrol B protects against cholestatic liver injury through pregnane X receptors. Br J Pharmacol. 2017;174:672-688. doi: 10.1111/bph.13729.

[49] Jiang YM, Wang Y, Tan HS, Yu T, Fan XM, Chen P, et al. Schisandrol B protects against acetaminophen-induced acute hepatotoxicity in mice via activation of the NRF2/ARE signaling pathway. Acta Pharmacol Sin. 2016;37(3):382-389. doi: 10.1038/aps.2015.120.

[50] Mao X, Gu C, Ren M, Chen D, Yu B, He J, et al. l-Isoleucine Administration Alleviates Rotavirus Infection and Immune Response in the Weaned Piglet Model. Front Immunol. 2018;9:1654. doi: 10.3389/fimmu.2018.01654.

[51] Gambardella J, Khondkar W, Morelli MB, Wang X, Santulli G, Trimarco V. Arginine and Endothelial Function. Biomedicines. 2020;8(8):277. doi: 10.3390/biomedicines8080277.

[52] Morris CR, Brown LAS, Reynolds M, Dampier CD, Lane PA, Watt A, et al. Impact of arginine therapy on mitochondrial function in children with sickle cell disease during vaso-occlusive pain. Blood. 2020;136(12):1402-1406. doi: 10.1182/blood.2019003672.

[53] Walocko FM, Eber AE, Keri JE, Al-Harbi MA, Nouri K. The role of nicotinamide in acne treatment. Dermatol Ther. 2017;30(5):10. doi: 10.1111/dth.12481.

[54] Buque A, Bloy N, Kroemer G, Galluzzi L. Possible mechanisms of cancer prevention by nicotinamide. Br J Pharmacol. 2021;178:2034-2040. doi: 10.1111/bph.15096.

[55] Nikas IP, Paschou SA, Ryu HS. The Role of Nicotinamide in Cancer Chemoprevention and Therapy. Biomolecules. 2020;10(3):477. doi: 10.3390/biom10030477.

[56] Cheng F, Zhou Y, Wang M, Guo C, Cao Z, Zhang R, et al. A review of pharmacological and pharmacokinetic properties of stachydrine. Pharmacol Res. 2020;155:104755. doi: 10.1016/j.phrs.2020.104755.

[57] Wang TA, Zhang XD, Guo XY, Xian SL, Lu YF. 3-bromopyruvate and sodium citrate target glycolysis, suppress survivin, and induce mitochondrial-mediated apoptosis in gastric cancer cells and inhibit gastric orthotopic transplantation tumor growth. Oncol Rep. 2016;35(3):1287-1296. doi: 10.3892/or.2015.4511.

[58] Ren JG, Seth P, Ye H, Guo K, Hanai JI, Husain Z, et al. Citrate Suppresses Tumor Growth in Multiple Models through Inhibition of Glycolysis, the Tricarboxylic Acid Cycle and the IGF-1R Pathway. Sci Rep. 2017;7:4537. doi: 10.1038/s41598-017-04626-4.

[59] Phillips R, Hanchanale VS, Myatt A, Somani B, Nabi G, Biyani CS. Citrate salts for preventing and treating calcium containing kidney stones in adults. Cochrane Database Syst Rev. 2015:D10057. doi: 10.1002/14651858.CD010057.pub2.

[60] Jiang T, Zhang L, Ding M, Li M. Protective Effect Of Vasicine Against Myocardial Infarction In Rats Via Modulation Of Oxidative Stress, Inflammation, And The PI3K/Akt Pathway. Drug Des Devel Ther. 2019;13:3773-3784. doi: 10.2147/DDDT.S220396.

[61] Rabelo TK, Guimarães AG, Oliveira MA, Gasparotto J, Serafini MR, de Souza Araújo AA, et al. Shikimic acid inhibits LPS-induced cellular pro-inflammatory cytokines and attenuates mechanical hyperalgesia in mice. Int Immunopharmacol. 2016;39:97-105. doi: 10.1016/j.intimp.2016.07.016.

[62] Eidi A, Mortazavi P, Tehrani ME, Rohani AH, Safi S. Hepatoprotective effects of pantothenic acid on carbon tetrachloride-induced toxicity in rats. EXCLI J 2012;11:748-759.
